# A Novel Tool Improves Existing Estimates of Recent Tuberculosis Transmission in Settings of Sparse Data Collection

**DOI:** 10.1371/journal.pone.0144137

**Published:** 2015-12-17

**Authors:** Parastu Kasaie, Barun Mathema, W. David Kelton, Andrew S. Azman, Jeff Pennington, David W. Dowdy

**Affiliations:** 1 Department of Epidemiology, Bloomberg School of Public Health, The Johns Hopkins University, Baltimore, MD, United States of America; 2 Department of Epidemiology, Mailman School of Public Health, Columbia University, New York, NY, United States of America; 3 Department of Operations, Business Analytics, and Information Systems, University of Cincinnati, Cincinnati, OH, United States of America; University of Cape Town, SOUTH AFRICA

## Abstract

In any setting, a proportion of incident active tuberculosis (TB) reflects recent transmission (“recent transmission proportion”), whereas the remainder represents reactivation. Appropriately estimating the recent transmission proportion has important implications for local TB control, but existing approaches have known biases, especially where data are incomplete. We constructed a stochastic individual-based model of a TB epidemic and designed a set of simulations (derivation set) to develop two regression-based tools for estimating the recent transmission proportion from five inputs: underlying TB incidence, sampling coverage, study duration, clustered proportion of observed cases, and proportion of observed clusters in the sample. We tested these tools on a set of unrelated simulations (validation set), and compared their performance against that of the traditional ‘*n-*1’ approach. In the validation set, the regression tools reduced the absolute estimation bias (difference between estimated and true recent transmission proportion) in the ‘*n-*1’ technique by a median [interquartile range] of 60% [9%, 82%] and 69% [30%, 87%]. The bias in the ‘*n-*1’ model was highly sensitive to underlying levels of study coverage and duration, and substantially underestimated the recent transmission proportion in settings of incomplete data coverage. By contrast, the regression models’ performance was more consistent across different epidemiological settings and study characteristics. We provide one of these regression models as a user-friendly, web-based tool. Novel tools can improve our ability to estimate the recent TB transmission proportion from data that are observable (or estimable) by public health practitioners with limited available molecular data.

## Introduction

Tuberculosis (TB) is unique among major infectious diseases in its ability to cause symptomatic and infectious (active) disease many years after transmission [1–3]. As a result, new cases of active TB represent a mix of recent transmission and remote infection (reactivation) [4–6]. Understanding the proportion of TB incidence due to recent transmission versus reactivation–a quantity that we term the “recent transmission proportion”–has important implications for implementation of TB control strategies, as different strategies (for example, contact investigation, improved diagnosis of active disease) may have greater epidemiological impact in settings of ongoing transmission [7–11], whereas other strategies (for example, preventive therapy) may be more relevant for settings where most active TB represents reactivation [12–14].

The traditional approach for estimating the recent transmission proportion is based on analysis of DNA fingerprints [15,16]. TB cases that are linked by recent transmission events should have similar DNA fingerprints (forming “clusters”), whereas those that represent reactivation will generally differ. Such fingerprinting methods are becoming increasingly important in assessing recent transmission in settings of both low [17–22] and high TB burden [23–25] for purposes of public health planning. While the molecular methods used for fingerprinting vary in their discriminatory power, and thus the epidemiological relevance of identified clusters, novel technologies (e.g., whole-genome sequencing) continue to reduce the probability of clustering by chance [26,27], thereby improving our ability to discriminate between reactivation and recent transmission.

**Fig 1:**
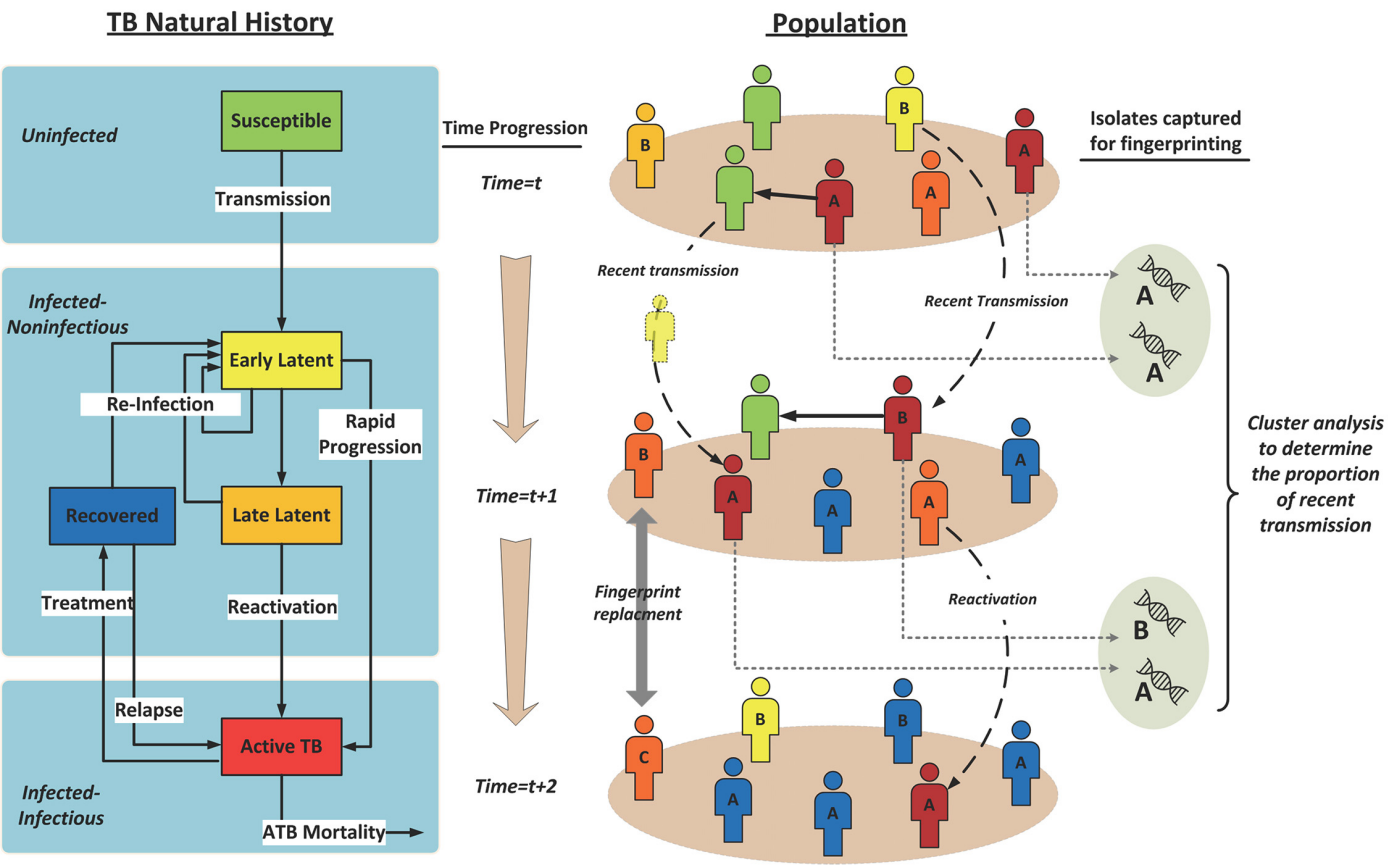
TB simulation overview. This figure illustrates our individual-based simulation model, following a hypothetical population for three consecutive (annual) time steps (Time = t, t+1, t+2). All infected individuals carry a single strain of TB (A, B, or C in this example). At each time step, three processes are modeled: 1. Transmission: upon successful contact, actively infected individuals can transmit the disease (marked by their strain type) to other people in the population. 2. Progression: other TB states are updated as shown in the left panel, including stabilization of latency, re-infection, diagnosis, and treatment, and relapse. Individuals who are diagnosed have their strain type recorded for analysis as they move from the active to the recovered state. 3. DNA fingerprint replacement: a random number of individuals in the late latency state are selected to carry new and unique fingerprints (strains), to maintain genetic diversity and account for processes such as mutation, migration, and infection from outside the population.

Unfortunately, technological advances in fingerprinting techniques have not been mirrored by analytical advances in estimating the recent transmission proportion [28],which is still frequently calculated using the simplistic assumption that, in each cluster, one case represents reactivation (the index) whereas all other cases represent recent transmission–an approach known as the ‘*n-*1’ method [5]. The ‘*n-*1’ method has several known biases [29–33] that limit its public health utility in settings where fingerprint data are incomplete or collected over a short time period, even when the fingerprinting technique itself is highly discriminatory. This is a broadly applicable problem in evaluating the dynamics of infectious diseases with a long transmission time scale (e.g., HIV, hepatitis C [34–36]), where molecular or genetic clustering data from population-based studies with limited coverage are often used to draw inference as to the proportion of transmission that occurs in a specific setting or on a given timescale. A less biased, user-friendly alternative for estimating the recent transmission proportion would therefore enable public health practitioners to maximize the utility of their molecular data. We therefore built a model of a generalized TB epidemic (Fig 1), and created a set of controlled simulation experiments (“*derivation set*”) to develop an improved tool for estimating the recent transmission proportion from molecular fingerprint data. We compared the performance of this tool against the traditional ‘*n*-1’ model, and validated the findings using an independent set of simulations (“*validation set*”). In order to illustrate the performance of this tool in a real setting, we also investigated its potential use on published data from Cape Town, South Africa [23].

## Methods

We constructed a stochastic, individual-based simulation model of a TB epidemic that incorporates elements of TB natural history, TB epidemiology, and hypothetical molecular epidemiological studies (Fig 1). We modeled a hypothetical population of 100,000 individuals with homogenous mixing structure, calibrating birth and death rates to preserve the mean population size over time. TB natural history is modeled at an individual level in five states: uninfected, early latent, late latent, active disease, and recovered (Fig 1 and Table 1) [37]. A complete description of the model is provided in Section A of the S1 Appendices.

**Table 1:** Model parameters.

Parameter	Value / [Range]	Source/Description
**Natural History:**		
Cumulative proportion of TB infections that progress to active disease over five years	13.8%	[38]
Mortality rate from untreated active TB	0.12 per year	[39]
Rate of successful diagnosis and treatment of active TB	0.9 per year	Calibrated to provide prevalence/incidence ratio of 1.4, accounting for people on treatment [40]
Rate of relapse to active TB during the first two years after treatment	0.06 per year	Calibrated to provide 15% annual cumulative incidence among previously treated individuals [40]
Proportion of TB re-infections that successfully replace a latently established strain	50%	[41–42]
**Setting-Specific:**		
Replacement rate	[0.5–10]x10-3 per year	Derivation set samples the following six values: (0.5, 1, 2, 3, 4, 5, 7, 10) × 10–3
Incidence rate	[100–450] per 100,000 per year	Derivation set samples values in increments of 50 (e.g., 100, 150, 200, etc.); calibrated by changing the contact rate at each level of the replacement rate above.
Duration of time over which isolates are collected	[2–20] years	Derivation set samples values in increments of 2
Proportion of the population contributing isolates for fingerprinting	[20–100]%	Derivation set samples values in increments of 20%

To capture the clustering dynamics of TB strains, we defined individual TB genotypes and modeled each transmission as resulting in an infection with a strain that, if isolated and fingerprinted, could be linked to the source case. Reinfection may occur, with an individual’s simulated DNA fingerprint reflecting the most recent transmission event. Over time, the simulated closed population develops more strain homogeneity than observed in real populations where migration and bacterial polymorphisms introduce additional diversity. We thus instituted a “fingerprint replacement” process in which a random set of individuals with latent infection change mycobacterial genotypes each year [37], representing a combination of infection from individuals outside the population, immigration/emigration, and bacterial evolution [43,44]. To minimize potential bias of this approach (to maintain long-term diversity in each simulation, whereas clustering is measured in the short term), we performed this procedure only on individuals who were infected more than five years previously. The replacement rate was calibrated to observed levels of TB genetic diversity [28,45] (Section A.1. of the S1 Appendices).

### Simulation experiments

After calibration, we developed a “derivation” set of simulations designed to cover setting-specific variables at regular intervals (for example, sampling TB incidence in intervals of 50 per 100,000/year), with natural history parameters held fixed at their best estimated value. Simulation scenarios were characterized by level of disease incidence as well as underlying proportion of incidence due to recent transmission (calibrated via contact rate and annual reactivation rate parameters). Limiting the scope of our study to settings of medium-to-high TB burden, we let the incidence level vary over the range of 100 to 450 cases per 100,000 per year [46], covering the parameter space using fixed intervals. Once TB natural history parameters were established, we then simulated different data collection exercises, in which a given proportion of individuals diagnosed with active TB (the sampling coverage) was fingerprinted over a specified period of time (the sampling duration). From each simulated “fingerprinting dataset”, we calculated the corresponding number of clustered cases (*C*) and the number of clusters (*N*) that would be observed, assuming a fingerprinting technique of perfect discrimination. The traditional ‘*n-*1’ estimate of the recent transmission proportion can then be calculated as *(N-C)/SS*, where *SS* is the sample size (Section A.2 of the S1 Appendices). We compared this ‘*n-*1’ estimate with the actual (simulated) recent transmission proportion in each scenario to calculate the bias in the ‘*n-*1’ estimate. We defined the “estimation bias” as the absolute underestimation or overestimation of the true recent transmission proportion.

After calculating the *‘n-*1’ estimate and its bias, we used the simulated data in the derivation set to develop an improved estimator of the recent transmission proportion via multiple linear regression incorporating five input variables: incidence, duration, coverage, ratio of clustered cases in the sample (*c = C/SS*), and ratio of observed clusters in the sample (*n = N/SS*). We evaluated both a simple linear model with these five covariates, as well as a more comprehensive model in which all potential multiplicative interaction terms were considered; models with even greater detail did not provide significant improvement in fit (data not shown). Full regression equations are provided in Section C.1 of the S1 Appendices and as an online calculator (http://modeltb.org/recenttrans/).

To validate the performance of the regression models and study the sensitivity of results to variation of simulation parameters, we created a second set of simulations (the “validation set”) in which both natural history and setting-specific parameters were randomly sampled from wide underlying distributions (Section B of the S1 Appendices). The parameter space was sampled using a Latin hypercube design to generate a sample of 1000 scenarios, of which 518 corresponded to a incidence level of 100 to 450 per 100,000/year (as used in original analysis). Data-collection exercises using varying levels of study coverage and duration were subsequently modeled in each scenario. In each simulation, we calculated the recent transmission proportion with both the traditional ‘*n*-1’ method and the novel regression tools, and compared these to the “true” (simulated) value. We then calculated the bias in each estimation method as the difference between the estimated and the true value. We used partial rank correlation coefficients to evaluate associations between model input parameters and the resulting bias in each estimation method [47], and performed additional sensitivity analyses around the fingerprint replacement rate and in settings of very high incidence (sections D.2 and D.1 of the S1 Appendices).

Finally, to investigate the potential performance of suggested estimators in real settings, we investigated a representative case study from the literature of a genotyping study in Cape Town, South Africa, from 1993 to 1998 [23]. Calibrating the simulation model to the corresponding study settings, we compared the estimation bias of the ‘*n*-1’ approach and regression-based models for the recent transmission proportion. Due to uncertainty in estimating the underlying TB incidence from available data on notifications, we considered two scenarios using a high case detection ratio (percentage of incident TB cases that are notified) of 90% similar to that assumed in the simulation derivation set (scenario 1), and a lower value equal to the current estimate of 62% for South Africa (scenario 2). Under these scenarios, we calibrated the replacement rate to produce similar measures of clustering as were observed in the original study (Section E of the S1 Appendices).

## Results

### Regression-based Tool Performance

**Fig 2:**
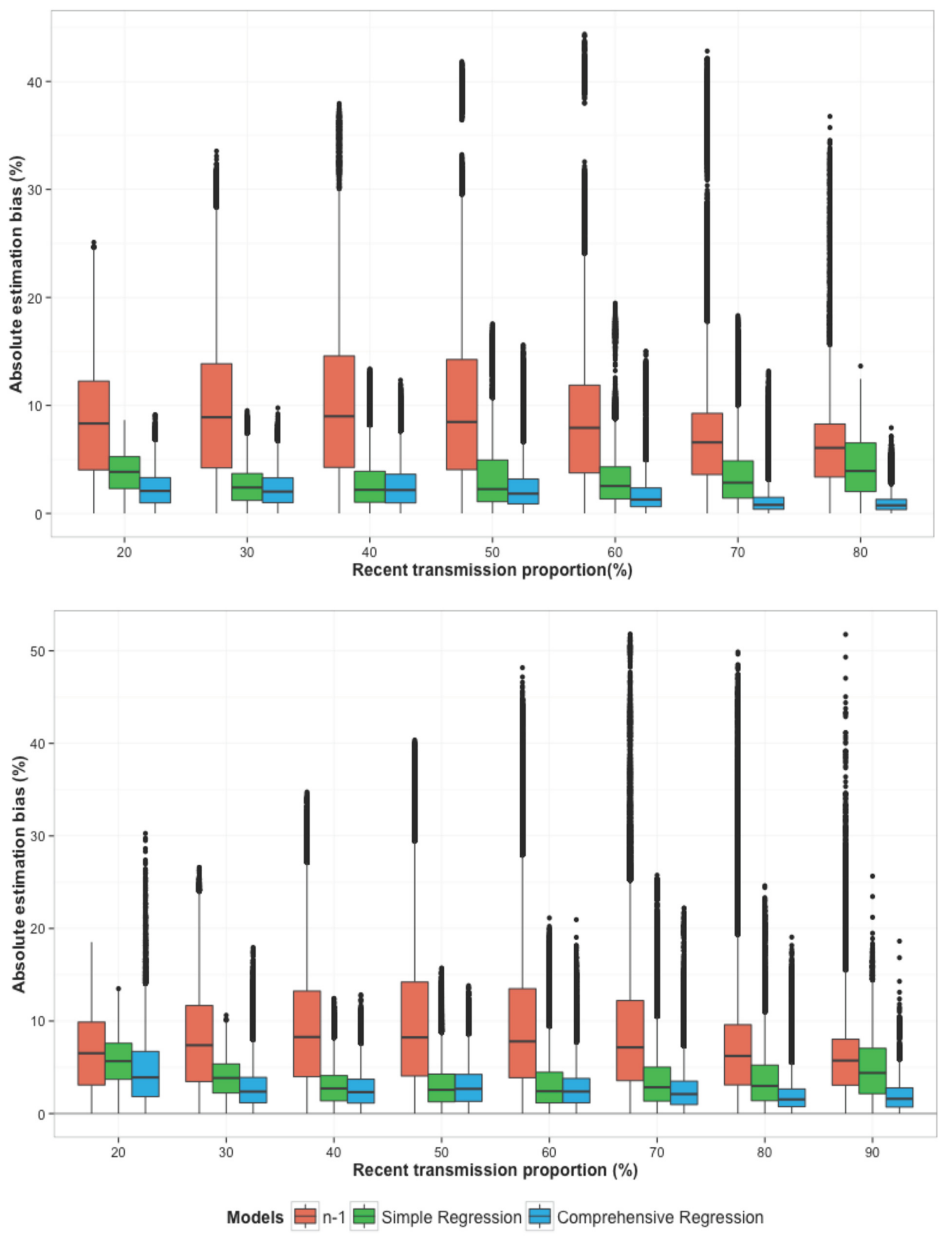
Absolute bias in estimates of the TB recent transmission proportion, comparing the ‘n-1’ method to novel regression-based tools in the derivation set (top) and validation set (bottom). The y-axis presents the absolute estimation bias [|estimated value – true value| × 100] in the proportion of incident active TB due to recent transmission (“recent transmission proportion”), and the x-axis denotes the recent transmission proportion (simulated) in each set of simulations. Estimates from the ‘*n-1*’ method are shown in red, and those from the simple and comprehensive regression tools are shown in green and blue, respectively. Boxes show the interquartile range of values from all simulations, and “whiskers” show the 95% confidence intervals, such that narrower boxes correspond to more precise (reproducible) estimates. The ‘n-1’ model tends to provide less accurate and precise estimates of recent transmission proportion (wide red bars) across all settings as compared to the simple and comprehensive regression-based models.

In the derivation set of simulation experiments, which sampled key parameters (TB incidence and replacement rate) at regular intervals (Table 1), the median [interquartile range] absolute bias in the estimated recent transmission proportion was 7.8% [3.9%, 12.3%] with the ‘*n-*1’ method (Fig 2- top graph). In other words, the median absolute difference between the recent transmission proportion as actually simulated versus as estimated by the ‘*n-*1‘ method was 8% (for example, 50% vs. 58%, or 75% vs. 83%). The simple regression model reduced this bias to 3.0% [1.4%, 4.6%], equivalent to a 65% [20%, 80%] reduction in the estimation bias of the ‘*n-*1’ method, and the comprehensive regression model reduced the bias further to 1.5% [0.6%, 3.0%], or a 78% [58%, 94%] reduction. The statistical significance of improvements was further confirmed via A Wilcoxon signed-ranked test by comparing the absolute estimation bias in the “*n-1*” method with the regression models (p-value< 0.001). When applied to the validation set, similar results were seen: median absolute estimation bias of 7.4% [3.6%, 12.2%] with the ‘*n-*1’ method, 3.1% [1.5%, 4.9%] with the simple regression model (60% [9%, 82%] reduction in bias), and 2.3% [1.1%, 3.8%] with the comprehensive regression model (69% [30%, 87%] reduction in bias) (Fig 2-bottom graph).

**Fig 3:**
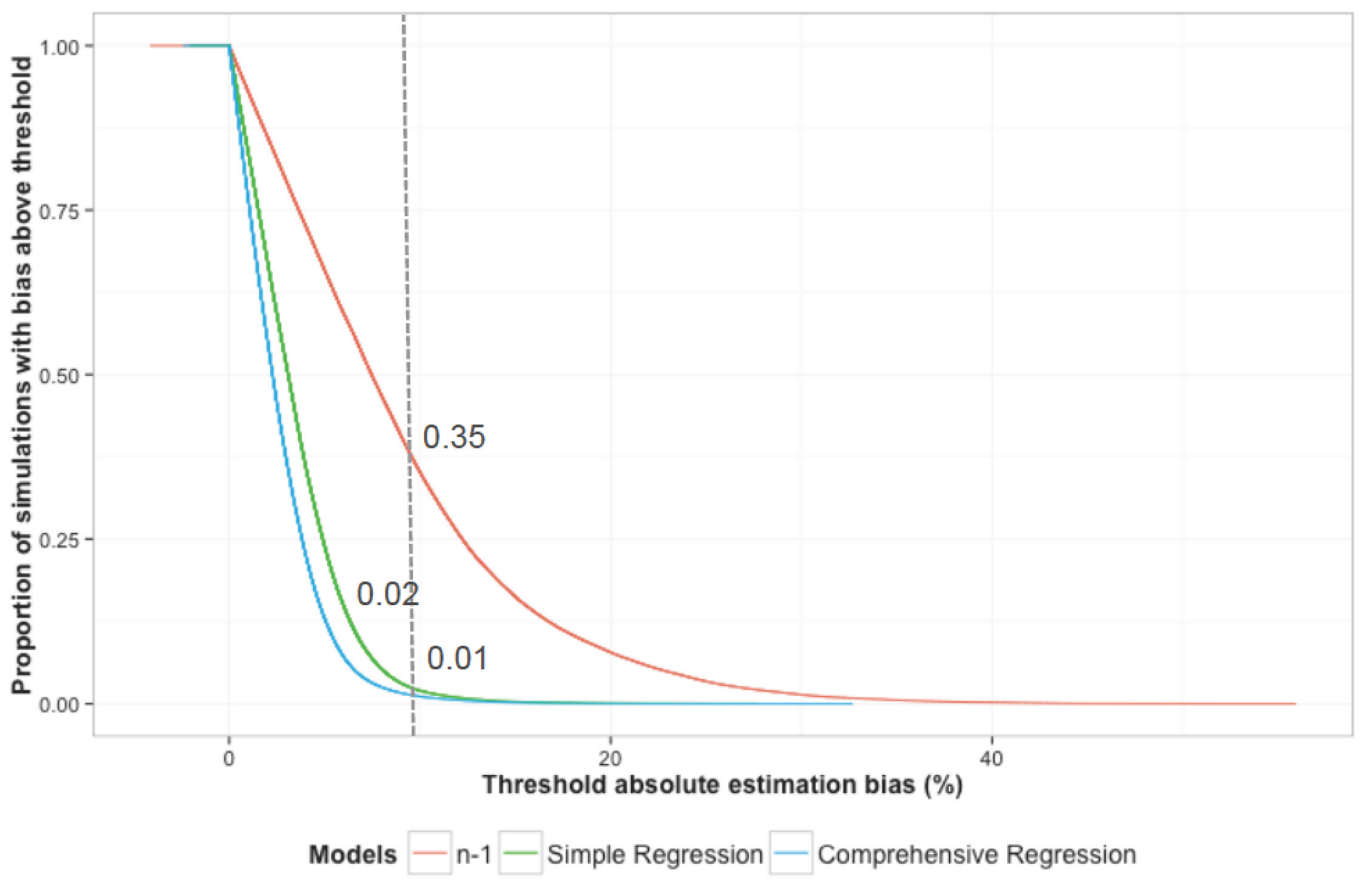
Absolute estimation bias in validation set, comparing the ‘n-1’ method to the regression-based models. The x-axis denotes the absolute level of estimation bias (abs[estimated value – true value]*100) in the proportion of incident active TB due to recent transmission (“recent transmission proportion”) across the validation set of simulations. The y-axis denotes the cumulative proportion of simulations with estimation bias greater than the threshold shown on the x-axis. For example, the vertical dotted line shows the proportion of simulations under each method that resulted in an absolute estimation bias of >10%: the ‘*n-1*’ method resulted in 10% or greater estimation bias in 35% of all simulations (red line), compared to 2% of all simulations with the simple regression model (green) and 1% of all simulations with the comprehensive regression model (blue).

As an outcome that might be relevant from a public health perspective, we measured the proportion of simulations in which the recent transmission proportion was over- or underestimated by 10% or more; 35% of simulations exceeded this threshold with the ‘*n-*1‘ method, versus 2% with the simple regression model, and 1% with the comprehensive regression model (Fig 3, grey dotted line). Thus, the *‘n-1’* method produced results with an absolute bias of more than 10% in over one-third of all simulated scenarios, whereas the regression tools resulted in such bias in less than one of every 50 simulations.

**Fig 4:**
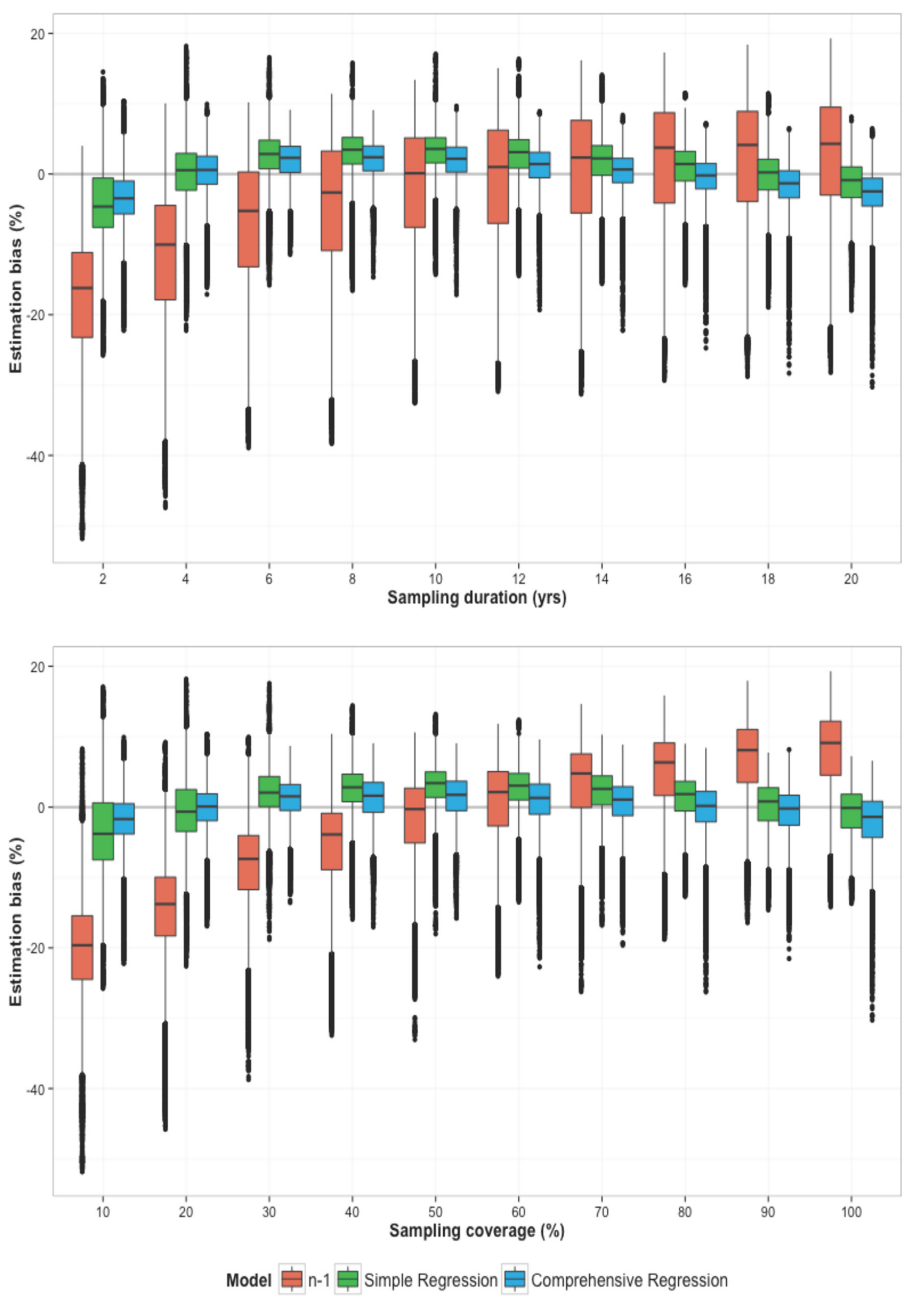
Estimation bias at different levels of study duration (top) and coverage (bottom), comparing the ‘n-1’ method to novel regression-based tools. The y-axis presents the (non-absolute) estimation bias [(estimated value – true value) × 100] in the proportion of incident active TB due to recent transmission (“recent transmission proportion”). Estimates from the ‘n-1’ method are shown in red, and those from the simple and comprehensive regression tools are shown in green and blue, respectively. Boxes show the interquartile range of values from all simulations, and “whiskers” show the 95% confidence intervals, such that narrower boxes correspond to more precise (reproducible) estimates. The ‘*n-1*’ model tends to underestimate the recent transmission proportion at low levels of sample coverage (<50%) and study duration (<10 years), and begins to overestimate the recent transmission proportion as coverage and duration are increased. The regression models are fairly robust to variation of study characteristics, providing more accurate and precise estimates of recent transmission proportion, especially in settings of incomplete coverage and short study duration.

Moreover, the ‘*n-*1‘ method’s accuracy in estimating the recent transmission proportion was highly sensitive to underlying levels of sampling coverage and duration (Fig 4). Specifically, fingerprinting studies with low coverage or short duration were more likely to generate underestimates of the recent transmission proportion, as cases in the same transmission chain were often miscategorised as non-clustered. This pattern is evident in the left side of Fig 4, for sampling duration of less than 10 years (upper panel) or sampling coverage of less than 60% (lower panel), where the ‘*n-*1’ approach (in red bars) increasingly underestimates the recent transmission proportion as data become more incomplete.

### Sensitivity Analysis

In analyses of partial rank correlation coefficients, bias in the ‘*n-*1’ estimates was strongly and positively correlated with the duration (PRCC = 0.79) and coverage of fingerprint data (PRCC = 0.89). The regression models, on the other hand, provided more accurate and precise estimates of the recent transmission proportion in the setting of incomplete data collection. For example, among simulations with 30–40% population coverage of molecular data over a 2-to-4 year duration of data collection, the simple regression model had a median estimation bias of 0.2% [-4%, 4%], compared to a substantial underestimation of -18% [-21%, -15%] with the ‘*n-1*’ method. At 70–80% coverage for 10–12 years, by contrast, the median bias in the simple regression estimate was 4% [2.4%, 5.5%], not noticeably different from that of the *‘n-1’* method (5% [4%, 7%]). These findings were consistent with the results of a large-scale whole-genome sequencing study in the Karonga district of Malawi [48], in which a sample of TB cases over 15 years with an approximate coverage of 50% resulted an estimate of 38% for the recent transmission proportion, whereas the simple regression estimate was about 32% (further details in Section C.4 of the S1 Appendices). PRCCs for correlation between the simple model output and study duration/coverage were -0.03 and 0.17, respectively (Section C.3 and C.4 of the S1 Appendices).

Variation in the underlying fingerprint replacement rate for individuals with latent TB infection influenced the performance of models, but it did not change the relative performance of the regression-based models versus the ‘*n*-1’ method. At higher fingerprint replacement rates (higher strain heterogeneity), the models tended to underestimate the recent transmission proportion [30,43], but the effect was similar both models (*‘n-1’* and regression), such that the regression models provided more precise estimates of recent transmission in all scenarios. Similarly, the models tended to overestimate the recent transmission proportion in settings with low fingerprint heterogeneity (low replacement rate) [49], but this effect was similar for both regression and *‘n-1’* techniques. Section D.1 of the S1 Appendices provides more detail. When we studied the performance of models in the remaining 419 simulations with an incidence higher than 450 per 100,000/year, the regression tools continued to outperform the ‘*n-*1’ method (Section D.2 of the S1 Appendices).

### Illustrative Case

**Fig 5:**
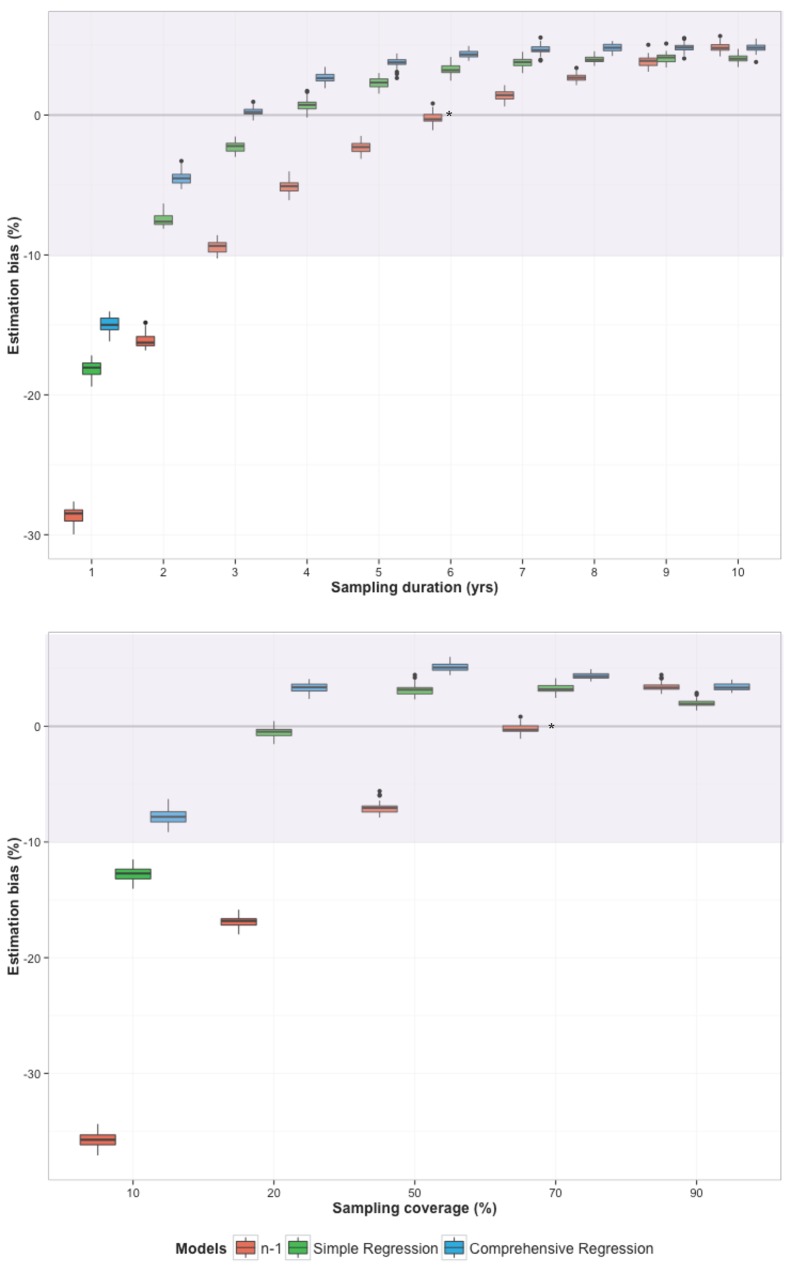
Estimation bias in the TB recent transmission proportion in an illustrative high burden case study, comparing the ‘n-1’ method to the regression-based models at different levels of sampling coverage. The top panel compares the estimation bias resulting from each model at a fixed sampling coverage of 73% (as estimated assuming a 90% case detection proportion, referred to as “Scenario 1” in the text), while varying the duration of data collection. The bottom panel present the results at a fixed study duration of 6 years (as reported in the original study), while varying the coverage of molecular fingerprints at the population level. The asterisk denotes the baseline scenario as reported in reference [23], at which all three methods accurately estimate the recent transmission proportion to within 5%. However, as study duration and population coverage decline, the performance of the ‘n-1’ method falls dramatically. At either a two-year study duration or a 20% population coverage of molecular data, the ‘*n-1*’ method underestimates the recent transmission proportion by 17% (second column of each figure), whereas both regression tools continue to estimate the recent transmission proportion with a bias of 7% or less. Boxes show the interquartile range of values from all simulations, and “whiskers” show the 95% confidence intervals, such that narrower boxes correspond to more precise (reproducible) estimates. Note that the three bars are jittered at each level of coverage/duration for clarity, but all three methods are performed under the same conditions.

In the original study (recreated by the calibrated simulation model as described in Section E of the S1 Appendices), the ‘*n*-1’ method provided a close estimate of the underlying recent transmission proportion (<1% bias, asterisk in Fig 5), due to reasonable completeness of data collection (e.g., sample duration of 6 years and coverage of 73% in scenario 1). This performance, however, deteriorated under the assumption that fingerprinting data might be more limited in corresponding programmatic settings. For example, if data were available for six years (as in the original study), but samples were collected only from 30% of diagnosed cases (instead of 73% in the original study), the median bias using the ‘*n*-1’ method inflated to -17% (Fig 5, lower panel, second column, red bars). Similarly, if the study duration was limited to two years, while maintaining the sampling coverage at 73% (per original study), the median bias using the *‘n*-1’ method was again -17% (Fig 5, upper panel, second column, red bars). In both of these cases, use of the regression tools rather than the *‘n-1’* method would have reduced this bias into the range of -7% to +3% (Fig 5, green and blue bars). Similar results were observed in scenario 2 (Fig N of the S1 Appendices), with the regression tools having similar performance to the *‘n-1’* method in settings of comprehensive data collection, but showing substantially less bias in settings where data collection was incomplete.

## Discussion

The recent transmission proportion is an important indicator of the degree to which observed TB incidence reflects ongoing disease transmission in a given population, with key policy implications including selection of appropriate interventions for disease control. We present here a set of two regression-based tools, the simpler of which is accessible via an easy-to-use web interface (http://modeltb.org/recenttrans/), and each of which removes 60–70% of the bias in estimating the recent transmission proportion in programmatic settings where molecular TB data may be incomplete or short-term in nature. Use of these tools may substantially advance our ability to link programmatic, often sparse molecular data on TB strain clustering to more accurate estimates of recent transmission and thus to more appropriate public health decision-making, without the need to conduct long and extensive molecular epidemiological studies where resources are limited. This meta-modeling technique using a combination of individual-based simulation and regression may also serve as a paradigm with broader application to using population-based (but sparse) molecular data to better understand infectious disease dynamics on longer time scales.

Previous studies have demonstrated the degree of bias in ‘*n-*1’ estimates [32], for example showing that clustering levels will underestimate recent transmission in settings of low data coverage. Our simulations further confirm these results, showing that gaps in sampling cause compensatory underestimates in the recent transmission proportion when using the ‘*n-*1’ method. Our model extends these earlier findings by providing an accessible tool that enables public health decision-makers to input estimates of study duration, sampling coverage, and TB incidence to obtain a more accurate estimate of the recent transmission proportion under local conditions. This new method is particularly relevant in settings of incomplete coverage of fingerprinting data, and accommodates the application of limited data to programmatic decision-making. Thus, a public health system in a high-burden country with no existing molecular repository may now be able to obtain reasonably accurate estimates of the recent transmission proportion by fingerprinting only a few years of data (either retrospective analysis of existing isolates or prospective collection of isolates over two to three years), and without the need for full population coverage (as long as the sample is representative of an underlying population). As an example, we studied the application of these regression tools in an epidemiological setting discussed in the literature [23] and evaluated the results assuming different levels of access to molecular fingerprinting data. Specifically, we found that, while both the regression tools and ‘*n-1’* method performed well in the setting of complete data availability, the regression tools markedly reduced bias in estimating the recent transmission proportion when data were incomplete (due either to short duration or low sampling coverage). These tools may therefore enable policy decisions that are more data-driven in settings of high TB burden, even when molecular fingerprint data are relatively sparse.

As with any other modeling study, our analysis has certain limitations. First, to retain strain diversity over time, we implemented an artificial “fingerprint replacement” procedure to capture uncertain patterns of migration, mutation rates, and exogenous infection over time. Our simulations may therefore underestimate the true recent transmission proportion in settings where mutation, migration, and mixing with external populations occur at a high rate, and overestimate the recent transmission proportion in isolated populations where such replacement rarely occurs. Importantly, we varied this “replacement” rate over a wide range in sensitivity analysis and did not see any substantial impact on our results. Second, because of difficulties in setting up appropriately representative closed-population simulations in low-burden settings where the majority of incident TB is imported, we restricted our model to moderate-to-high burden settings. These results should therefore be generalized to lower-burden settings only with caution, and future simulation efforts may be useful in developing a tool that is more appropriate for settings in which immigration drives the majority of incident TB. Third, as our aim was to provide a generalizable, transparent, user-friendly platform, we excluded complexities such as age structure, HIV coinfection, and heterogeneous mixing. Prior studies have suggested that these heterogeneities may affect the estimates of clustering (and therefore recent transmission proportion) in different ways (for example, underestimating clustering among younger individuals and overestimating clustering in older individuals) [33,43,50]. Future efforts could incorporate these assumptions into more complex models to evaluate the residual amount of bias introduced by such simplifications (which should apply equally to the regression and *‘n-1’* approaches). Finally, since our goal here was to generate a method that more accurately represents the truth (especially as the resolution of genotyping data is anticipated to improve over time), we assumed a genotyping method with perfect resolution between strains. To the extent that less-discriminatory methods are used for fingerprinting, estimates of clustering from all methods are likely to be positively biased, resulting in overestimates of the recent transmission proportion.

In summary, we have created novel tools, using regression and an individual-based simulation model, to better estimate the proportion of incident TB due to recent transmission in high-burden settings. These tools remove 60–70% of the bias intrinsic to the most commonly used method at present (the ‘*n-*1’ method), and the simple tool is easily accessible to epidemiologists and public health officials via a web-based user interface. Such approaches may have broader applicability to the estimation of clustered transmission of other infectious diseases as well. As we seek to accelerate progress in the fight against these diseases worldwide, better estimates of the recent transmission proportion in subpopulations will be critical to developing evidence-based public-health approaches that appropriately target those hotspots of recent transmission. For example, more accurate estimates of the recent progression proportion could assist local-level officials in deciding whether to allocate resources to interventions (e.g., contact investigation, improved diagnosis, and case finding) more appropriate for epidemics driven by recent transmission, or to those (e.g., preventive therapy) more targeted toward preventing reactivation. Tools such as these can help guide decision-makers to develop more effective policies and interventions in settings where data are often incomplete and long studies are impractical.

## Supporting Information

S1 Appendices (DOCX)
