# Supplementary material for: A Novel Tool Improves Existing Estimates of Recent Tuberculosis Transmission in Settings of Sparse Data Collection
Source: PLoS One. 2015 Dec 17;10(12):e0144137. doi: 10.1371/journal.pone.0144137 (PMC4683006; doi:10.1371/journal.pone.0144137)
Supplement: S1 Appendices — (DOCX) [file pone.0144137.s001.docx]

**S1. Appendices**

**A novel tool improves existing estimates of recent tuberculosis transmission in settings of sparse data collection**

**Authors:** Parastu Kasaie^1^, Barun Mathema^2^, W. David Kelton^3^, Andrew S. Azman^1^, Jeff Pennington^1^, and David W. Dowdy^1^

**Author Affiliations: The Johns Hopkins University, Baltimore, MD, 21205 (P. Kasaie, A.S. Azman, J. Pennington, D.W. Dowdy); Columbia University, New York, NY, 10032 (B. Mathema); University of Cincinnati, Cincinnati, OH, 45221 (W.D. Kelton)**

# Simulation model of TB: model design and experimental framework

Our stochastic individual-based simulation model represents the dynamics of TB transmission and strain clustering over time. We modeled a hypothetical population of 100,000 individuals (agents) with homogenous mixing structure modeled as a Poisson process with a mean contact rate that is calibrated to provide a specific underlying incidence level. Population dynamics are modeled through an age-structured natural mortality process and a random-birth process calibrated to preserve the mean population size. We constructed simulation experiments over moderate- to high-TB burden epidemiological settings (e.g., TB incidence), experimented with varying data-gathering characteristics (e.g., duration and sampling fraction of all culture-positive cases with genotyping data), and used these simulation experiments to develop regression-based tools for rapid calculation of the recent transmission proportion, as a function of known variables and sampling results.

The simulation model is programed in C++ and the code is accessible via https://github.com/pkasaie/TB_RecentTransmissionProportion.git.

**Model Design**

TB natural history is modeled at an individual level in five states: uninfected, early latent, late latent, active disease, and recovered (Figure 1 in the main manuscript). Early infection lasts for five years after successful contact/transmission, with each subsequent year carrying a lower risk of progression to active disease [1]. If active disease does not develop, individuals enter a late latent state carrying a persistent small risk of reactivation. Upon progression from infection (early or late latent) to active TB, individuals become increasingly infectious over nine months, after which infectiousness is assumed to stabilize [2]. The rate of diagnosis and treatment is calibrated to provide an average disease duration of 11 months [3]. Successful diagnosis and treatment results in immediate transition to a non*-*infectious state, but a percentage of these individuals are lost to follow-up or relapse to active TB within two years.

To capture the clustering dynamics of TB strains, we define TB genotypes at an individual level and model each transmission as resulting in an infection with a strain that, if isolated and fingerprinted, could be linked to the source case. Reinfection is possible, with an individual’s simulated DNA fingerprint reflecting the most recent transmission event if reinfection is “successful.” This results in a population that, over time, has less strain diversity than that observed in real populations where migration of individuals and bacterial polymorphisms introduce additional diversity. We thus institute an artificial “fingerprint replacement” process in which we select a random set of individuals in the late latent state every year and replace their genotype with one that, if active TB disease were to develop, would be sufficiently distinct (unlinkable to other strains in the population) [4]. This represents a combination of infection from individuals outside the population, immigration/emigration, and bacterial evolution [5,6]. To minimize the potential bias of this approach (to maintain long-term diversity in each simulation, whereas clustering is measured in the short term), we perform this procedure only on individuals in the late latent state. The annual proportion of individuals whose strains are replaced in this fashion (approximately 10%) is calibrated such that the number of unique strains is maintained at around 70% of the number of people infected with TB, as reported in studies of large populations using highly discriminatory methods in the literature [7,8].

**Simulation Experiments**

We sought to develop a simple rule for estimating the contribution of recent transmission to TB incidence using observable (or estimable) information from available molecular fingerprint data. To that end, we performed a set of simulations (the “derivation set”) to sample defined values of TB incidence, TB replacement rate, duration of fingerprinting data collection, and population coverage of fingerprint data, as shown in Table 1 of the main manuscript*.* Simulation scenarios are characterized by the incidence of active TB and the proportion of incidence that reflects recent transmission (“recent transmission proportion”). These scenarios are calibrated by varying parameters that correspond to individuals’ contact rate and annual replacement rate. Due to uncertainty regarding the annual rate of replacement, we explored a wide range of values for this parameter (using eight fixed levels as noted in Table 1 of the main manuscript). At each level, we calibrated the average incidence (to eight fixed levels) via the contact rate parameter. Once the TB natural-history parameters were established, we then simulated different data collection exercises, in which a given proportion of diagnosed individuals with active TB (the sampling coverage) is sampled, and the corresponding TB isolates are fingerprinted, over a specified period of time (the sampling duration). From each “fingerprinting dataset” simulated in this fashion, we calculated the corresponding number of clustered cases (*C*) and the number of clusters (*N*) that would be observed. The traditional ‘*n-*1’ estimate of the recent transmission proportion can then be calculated as *(N-C)/SS,* where *SS* is the sample size. We compared this ‘*n-*1’ estimate with the actual recent transmission proportion in each simulated scenario to calculate the extent of bias in the ‘*n-*1’ estimate in each setting, i.e., underestimation or overestimation of the true value of recent transmission proportion.

After calculating the *‘n-*1’ estimate and its bias, we used the simulated data in the derivation set to develop an improved estimator of the recent transmission proportion. For this purpose, we generated 224,000 observations (50 data-collection exercises in each of 64 simulation scenarios replicated 70 times; see Section 2 for more detail) and used multiple linear regression to predict the recent transmission proportion as a function of five input variables: incidence, duration, coverage, ratio of clustered cases in the sample (*c*), and ratio of observed clusters in the sample (*n*). (Thus, *c* = *C*/*SS* and *n* = *N*/*SS*.) We evaluated both a simple linear model with these five covariates, as well as a more comprehensive model in which all potential multiplicative interaction terms were considered and removed in stepwise fashion if not significantly associated with the outcome; models with even greater detail did not provide significant improvement in fit and therefore were not considered (data not shown). Full regression equations are provided in Section .3 and with the online calculator (http://modeltb.org/recenttrans/).

To validate the performance of the regression models and study the sensitivity of results to variation of simulation parameters, we created a second set of simulations (the “validation set”) in which all setting-specific parameters (incidence, replacement rate, study duration, and coverage) were randomly sampled from their original range assuming a uniform distribution, and all natural history parameters were varied by -/+50% of their original values assuming a beta distribution, as described in Section .2.2. The parameter space was sampled using a Latin hypercube design to generate a sample of 1000 candidate scenarios, out of which 518 corresponded to an incidence level of 100 to 450 per 100,000/year (as used in original analysis). Data-collection exercises using varying levels of study coverage and duration were subsequently modeled in each scenario resulting in 518,000 observations (20 replications of 50 data-collection tests in each experiment). In these simulations, we compared the bias in the traditional ‘*n-*1’ estimate of the recent transmission proportion to the bias in the regression-based estimates, using the actual simulated value as the “gold standard.” We used partial rank correlation coefficients to evaluate associations between model input parameters and the resulting bias in each estimation method, holding other input parameter values constant [9]. Further sensitivity analyses were performed, in which we varied the underlying fingerprint replacement rate and evaluated model performance in high burden settings (see Section .4). Finally, we adopted a representative genotyping study from the literature [10], and investigated the performance of suggested models in this case study (see Section .5).

## Calibrating the diversity of TB strains

The underlying simulation model takes a previously published representation of TB natural history [4]. We refine the previous definition of ‘migration’, which affects all individuals in the population, to a ‘fingerprint replacement’ procedure, restricted to only those individuals in the late latency period.

To calibrate the fingerprint replacement rate, we consider the *Ratio of Circulating TB Strains* (RCS) as a measure of TB strain diversity over time, where:

$$RCS\left( t \right)=\frac{\text{Number of circulating strains at time} t}{\text{Non-susceptible population size at time} t}$$

RCS levels range between 0 and 1, where a value of 1 corresponds to a population with no DNA clusters such that every infected person (whether latent, active, or recovered) carries a unique strain of TB. The average RCS level is influenced by the underlying annual fingerprint replacement rate (Figure A).

Figure A: Relationship between the annual fingerprint-replacement rate and the simulated RCS level as a baseline. The baseline scenario corresponds to a scenario with incidence of 200 cases per 100,000/year and an underlying replacement rate of 0.001 per year. The RCS levels are computed as average annual values at equilibrium (year 200 to 300).

The diversity of circulating TB strain types among populations is a matter of uncertainty, and the only available source of data are molecular studies of TB DNA fingerprints in various settings. In order to estimate the average ratio of circulating strains in populations, we compute the ratio of unique strain types in a number of such studies as reviewed by [7] and rely on this ratio as a crude (lower-bound) estimator of the underlying ratio of circulating strains. The average ratio of unique strains among all studies is about 65% as shown in Figure B. Moreover, we refer to [8] for a similar estimate of 70% for the ratio of circulating strains among the infected US population. Accordingly, we choose an approximate level of 60% as the ratio of circulating TB strains at baseline, and calibrate the annual fingerprint-replacement rate in the simulation model accordingly: a 10%/year fingerprint replacement rate provides a 60% ratio of circulating TB strains.

Figure B: Ratio of unique TB strains observed in previous molecular epidemiological studies of TB.

## Simulation procedure

The individual-based simulation models the long-term dynamics of disease diffusion and strain clustering over time. The model takes two input parameters for initializing each simulation scenario, *S(i,r)*, corresponding to the TB incidence rate (*i*) and underlying replacement rate (*r*). The incidence level is calibrated via the contact rate parameter in the simulation model. The simulation runs in discrete time steps representing months, and the output is reported at the end of each year (*t*= 1, …, *T* where *T* is the simulation time horizon). All simulation scenarios, *S(i,r)*, are repeated for *R* replications to provide a 95% confidence-interval half-width of at worst 1 person around the expected incidence level.

Each simulation scenario is used to generate a virtual repository of DNA fingerprints from all diagnosed cases over time. To this end, we record the DNA fingerprints of all diagnosed TB cases at the end of each year, and update the corresponding DNA repository for this scenario (Figure C). The DNA repositories are subsequently used in the ‘data collection & analysis’ procedure for analyzing the cluster dynamics of TB strains and estimating the recent transmission proportion in each scenario.


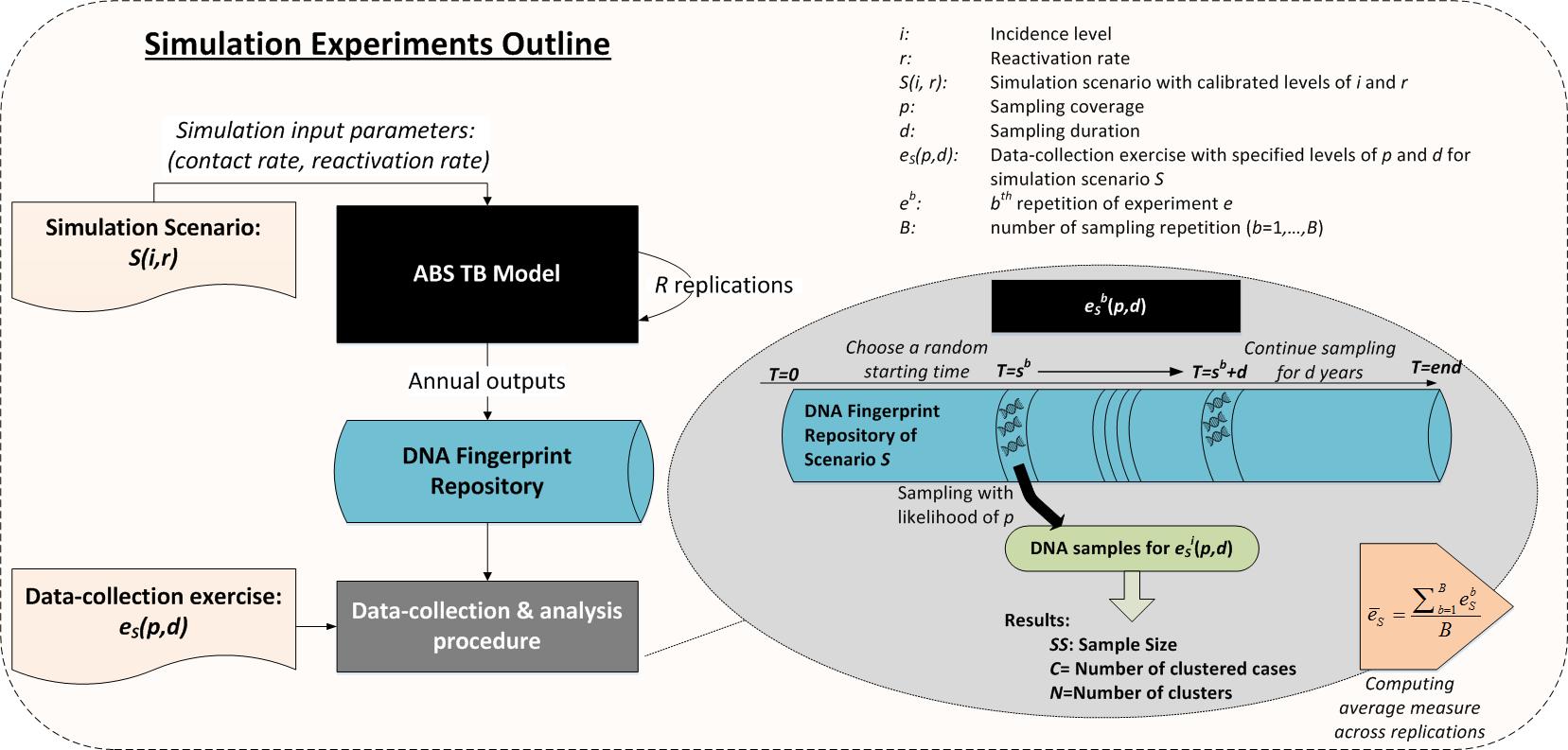


Figure C: Simulation experiment outline. The agent-based simulation (ABS) model is used to create a simulation scenario corresponding to *S*(*i,r*) and generating virtual repositories of TB strains (DNA fingerprints) from all diagnosed patients over time. This repository will be subsequently sampled through a data-collection exercise *e*(*p,d*). The subsequent sample is analyzed for clustering patterns of DNA fingerprints and estimating the recent transmission proportion through the ‘*n-*1’ approach.

**Data-collection & analysis procedure:**

Each ‘data-collection exercise’, *e_S_(p,d)* is associated with specified levels of study coverage (*p*) and duration (*d*), and is conducted over a DNA repository corresponding to simulation scenario *S*. Each experiment is carried out over a random selection from the DNA repository, starting from randomly selected year *s*, and continuing for *d* consecutive years. In each year, DNA isolates are sampled with an ascertainment probability of *p* for each individual. The final sample is analyzed for clustering patterns, and we report three values, the sample size (*SS*), the number of clustered cases (*C*), and the number of observed clusters (*N*). The reported values can be used to estimate the clustering level using the *‘n-*1*’* method (‘*n*-1’ estimate = (*C-N)/SS*). To increase the precision of reported data-collection exercises for each scenario, we repeat each data collection exercise *B* times, each time using a random starting point *s*, to ensure an unbiased observation of clustering dynamics over time. The value of *B* is set to 50 repetitions in order to increase the precision of averaged reported results. At each repetition, the sampling start time *s* is randomly chosen from the beginning of simulation steady state (taken to be year 101) up to year *T-d*. Figure C shows the conceptual model of simulation experiments for this analysis.

# Model calibration

## Derivation set

We constructed a set of simulations (the “derivation set”) to represent a wide range of epidemiological settings with regard to TB incidence and proportion of incidence due to recent transmission. Due to the intrinsic dynamics of several parameters in the TB natural history model (Figure D), the subsequent recent transmission proportion in each simulation scenario is affected by several factors, including the contact rate, and the underlying value of primary infection- and replacement-rate parameters. For calibration purposes, we assume a fixed value of the primary infection rate over time, and use a combination of contact rate and replacement rate parameters to calibrate the corresponding levels of TB incidence and recent transmission proportion. Considering a setting of medium to high TB burden, we assume eight levels of incidence uniformly distributed across the range of [100, 450] per 100,000/year. Due to uncertainty regarding the true value of the annual replacement rate, we explore a wide range of variation (ranging from 0.5 × 10^-3^ to 10 × 10^-3^) for this parameter. Using trial and error, we chose six levels of the replacement rate to provide a subsequent uniform distribution of the recent transmission proportion among various incidence levels, i.e., at each level of the replacement rate, we use the mean contact parameter to calibrate the average TB incidence the pre-specified incidence levels, and compute the estimated recent transmission proportion from that scenario. The scenarios are replicated R=70 times to provide a 95% confidence-interval half-width interval of at worst 1 (person) around the expected incidence level. This results in (8*8*70=4480) independent observations on the simulation model. Figure D shows the estimated levels of incidence and recent transmission proportion across all simulation scenarios in the derivation set.


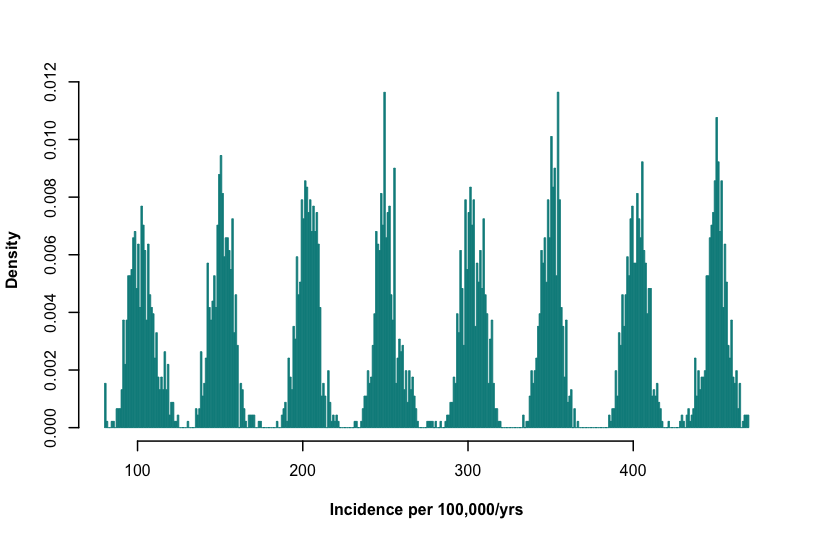

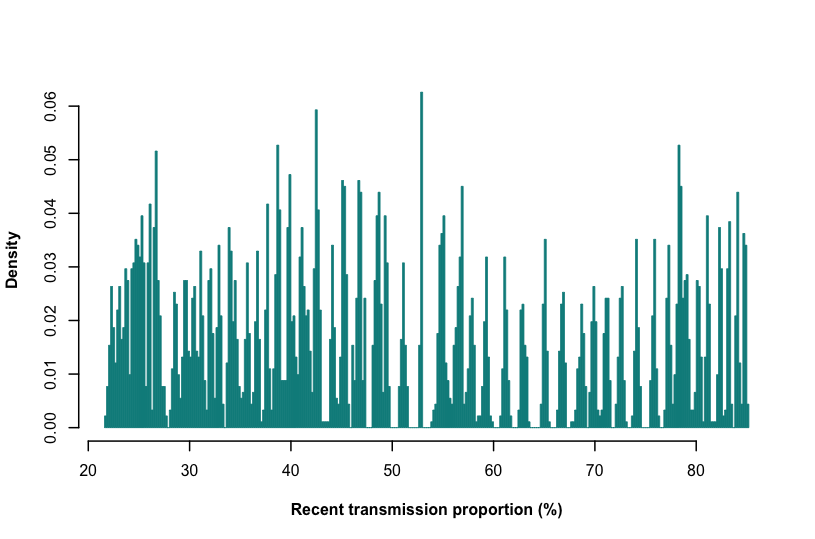


Figure D: Estimated levels of average TB incidence and recent transmission proportion in the derivation set of simulations.

## Validation set

To check the performance of the regression model in a generalized setting, and as a means for sensitivity analysis of results to variation of simulation parameters, we designed a second set of random simulations in the validation set.

**Natural-history parameters:**

We assume an underlying beta distribution, Beta(a,b), for initially fixed model parameters, arbitrarily choosing alpha (shape parameter) =4 and fitting b (using the distribution mean) to the corresponding parameter value in the derivation set. We allowed each parameter to vary within a 50% interval from its original value, and sampled the fitted distribution within this range, e.g., sampling the fitted distribution to parameter x from [0.5*m_x_, 1.5*m_x_] where m_x_ is the original (mean) level of parameter x in the derivation set. We then use a Latin hypercube design to generate parallel random samples of all model parameters from their corresponding fitted distributions and within the allowable variation range.

**Setting-specific parameters:**

The replacement rate was randomly sampled from a uniform distribution covering its original range [0.5-10] × 10^-3^. Moreover, due to implicit dependency of the replacement rate and contact rate parameters in determining the level of incidence in each setting, we chose a feasible range of the contact rate parameter in relation to the replacement rate and sampled the contact parameter value uniformly within this range.

Subsequently, we generated 1000 random samples of all parameters and estimated the resulting levels of TB incidence (per 100,000 per year) in each scenario (Figure E).


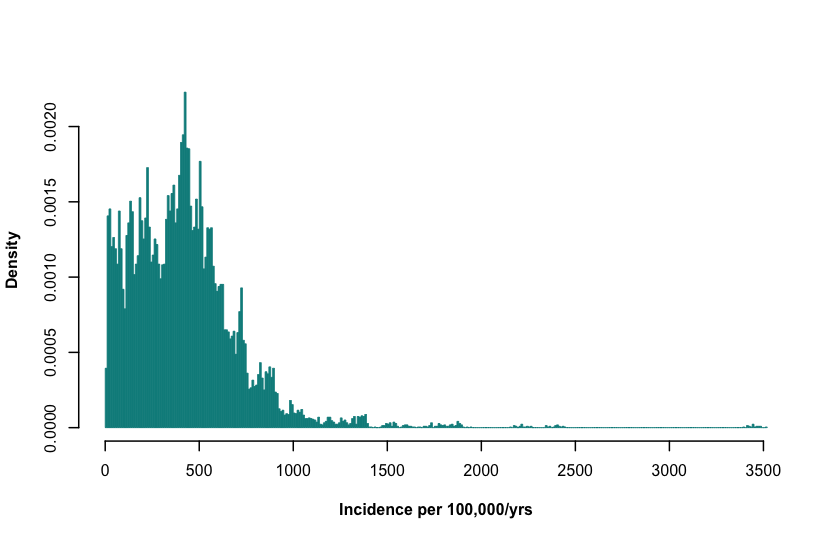


Figure E: Average TB incidence distribution in the initial random sample of 1000 simulations.

The initial random design (including 1000 scenarios) was then scanned and filtered for scenarios that corresponded to an incidence of 100 to 450 per 100,000/year in the simulation model, resulting in 518 scenarios in the validation set. We subsequently used the remaining high-incidence scenarios (419 scenarios with incidence greater than 450 per 100,000/year) in a sensitivity analysis to study the performance of the model in high-burden settings (see Section 4.1). Figure F shows the subsequent distribution of incidence and recent transmission proportions, as well as input parameter settings, in the validation set of scenarios.

**Data-collection exercises:**

Feasible scenarios were simulated for 20 replications, and corresponding DNA repositories were generated. At each scenario, a set of 50 random data-collection exercises was performed with experiment parameters (sampling duration and coverage) randomly chosen from their original range (as noted in Table 1 of the main paper). Figure G shows the range of variation for each parameter across the validation set.


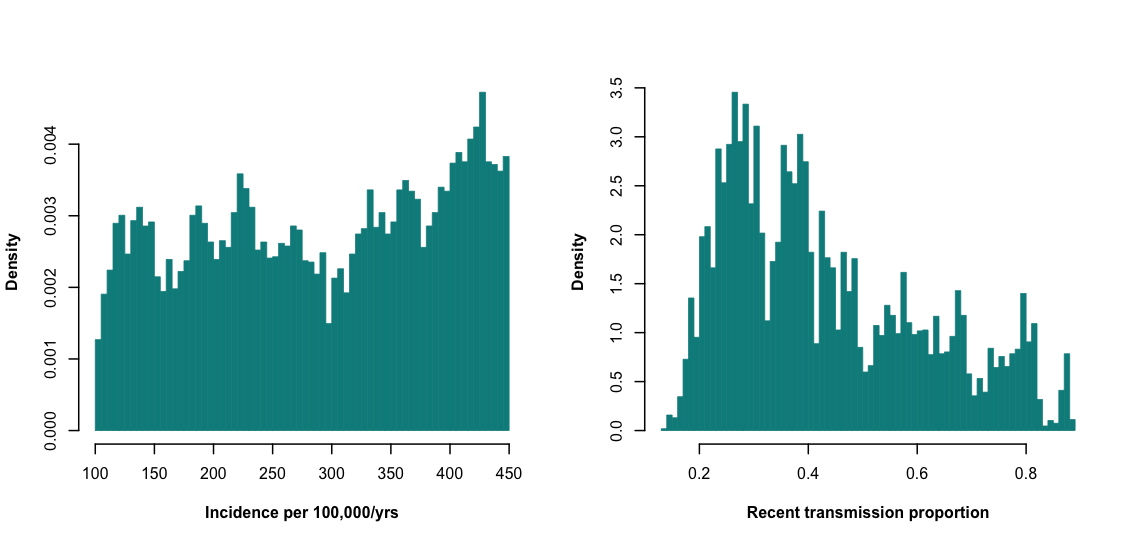


Figure F: Probability density distribution of average TB incidence and recent transmission proportion in the validation set.


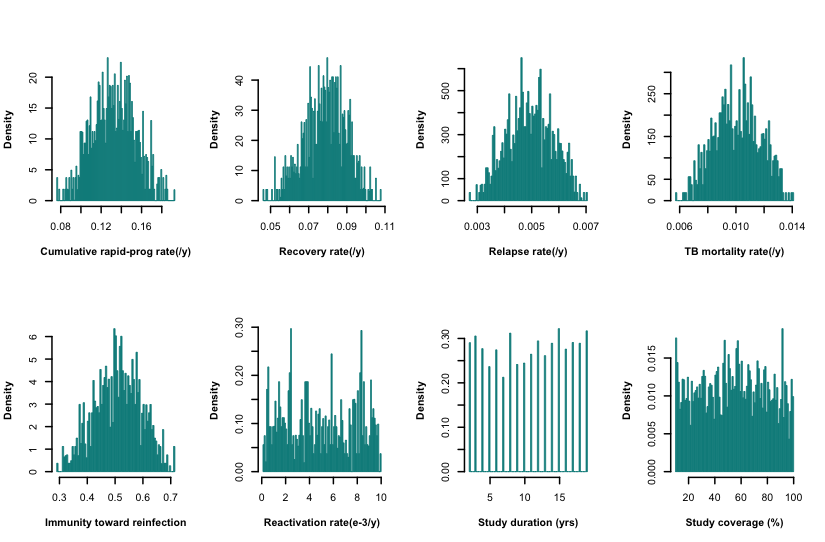


Figure G: Parameters’ variation in the validation set. These graphs show the probability density distribution of various epidemiological/study characteristics parameters used in the validation set of simulations. Natural-history parameters (first five graphs) are randomly sampled from a beta distribution calibrated to their original mean, while the setting-specific parameters (last three graphs) are uniformly sampled from their original range.

# Results

## Regression-based models

Each data collection exercise is characterized by a set of parameters describing the duration and coverage. From each “fingerprinting dataset” simulated in this fashion, we calculate the corresponding number of clustered cases (*C*) and the number of clusters (*N*) that would be observed. The traditional ‘*n-*1’ estimate of the recent transmission proportion can then be calculated as *(N-C)/SS*, where SS is the sample size. Subsequently, we compare this ‘*n-*1’ estimate with the actual recent transmission proportion in each simulated scenario to calculate the bias in the ‘*n-*1’ estimate in each setting (i.e., such bias can correspond to an underestimation or overestimation of the true value of the recent transmission proportion). We also use the simulated data to develop an improved estimator of the recent transmission proportion through two regression models.

Table A provides the complete formulation of each regression model for estimating the recent transmission proportion. Regression coefficients include the proportion of observed clustered cases in the sample *(c)*, the proportion of observed clusters in the sample (*n*), the sampling coverage (*p)*, the sampling duration (*d*), and the incidence (*i*)*,* the regression response is the true (simulated) level of the recent transmission proportion in each simulation scenario (derivation set).

Table A: Regression-Based Models

| **Simple regression model with main effects:** | | | | | |
| --- | --- | --- | --- | --- | --- |
| (Intercept) | *c* | *n* | *p* | *d* | *i* |
| 3.27E-01 | 9.18E-01 | -8.63E-01 | -2.67E-01 | -9.40E-03 | -2.73E-05 |
|  |  |  |  |  |  |
| **Comprehensive regression model with main effects and interactions**^[[1]](#footnote-1)^**:** | | | | | |
| (Intercept) | *c* | *n* | *p* | *d* | *i* |
| 2.37E-01 | 3.68E-01 | 6.67E-01 | 7.40E-02 | 2.72E-03 | 2.15E-04 |
| *c:n* | *c:p* | *n:p* | *c:d* | *n:d* | *p:d* |
| 1.46E+00 | 1.97E-01 | -2.74E+00 | 2.07E-02 | -3.66E-02 | -1.18E-01 |
| *c:i* | *n:i* | *p:i* | *d:i* | *c:n:p* | *c:n:d* |
| 3.38E-04 | -1.24E-03 | -8.39E-04 | -1.99E-05 | 2.97E-01 | -1.42E-01 |
| *c:p:d* | *n:p:d* | *c:n:i* | *c:p:i* | *n:p:i* | *c:d:i* |
| 9.84E-02 | 4.93E-01 | -1.91E-03 | 4.05E-04 | 5.71E-03 | -1.92E-05 |
| *n:d:i* | *p:d:i* | *c:n:p:d* | *c:n:p:i* | *c:n:d:i* | *c:p:d:i* |
| 1.25E-04 | 4.07E-05 | -3.16E-01 | -3.79E-03 | 8.34E-05 | -1.37E-05 |
| *n:p:d:i* | *c:n:p:d:i* |  |  |  |  |
| -4.65E-04 | 3.83E-04 |  |  |  |  |

The simple regression model has been publicly implemented in a user-friendly tool available at http://modeltb.org/recenttrans/. Users are prompted to provide the listed information in Table B, which will be used to evaluate the regression coefficient values.

Table B: List of required information for application of the regression tool.

| **Information requested from researcher** | **Symbol** | **Description** |
| --- | --- | --- |
| Population size | *POP* | Local population size |
| Annual incidence level per 100,000/year | *I* | If unknown, it can be estimated using the underlying case detection ratio and number of diagnosed TB cases^[[2]](#footnote-2)^. |
| Sample size | *SS* | Number of available DNA fingerprints in the sample |
| Sampling duration | *D* | Number of years the population was followed |
| Number of observed clusters of size 2 or greater in the sample | *N* | - |
| Number of observed clustered cases in the sample | *C* | - |
| **Regression coefficient** |  |  |
| Local incidence | *i* | *I*×*POP/*100,000 |
| Sampling duration | *d* | *D* |
| Sampling coverage | *p* | *SS/(*0.86×*i*×*d)* ^[[3]](#footnote-3)^ |
| Proportion of clusters in the sample | *n* | *N/SS* |
| Proportion of clustered cases in the sample | *c* | *C/SS* |
|  |  |  |

## Comparing model performance

Figure H compares the three models in terms of estimation bias in the derivation and validation set of simulations. The regression models provide more precise and accurate estimates of recent transmission proportion in comparison to the ‘n-1’ approach. The regression models’ performance is slightly worse in the validation set of scenarios, as expected. The initial difference in performance between the simple and comprehensive regression models (as apparent in the derivation set) is reduced in the validation set due to a higher degree of overfitting in the comprehensive regression model.


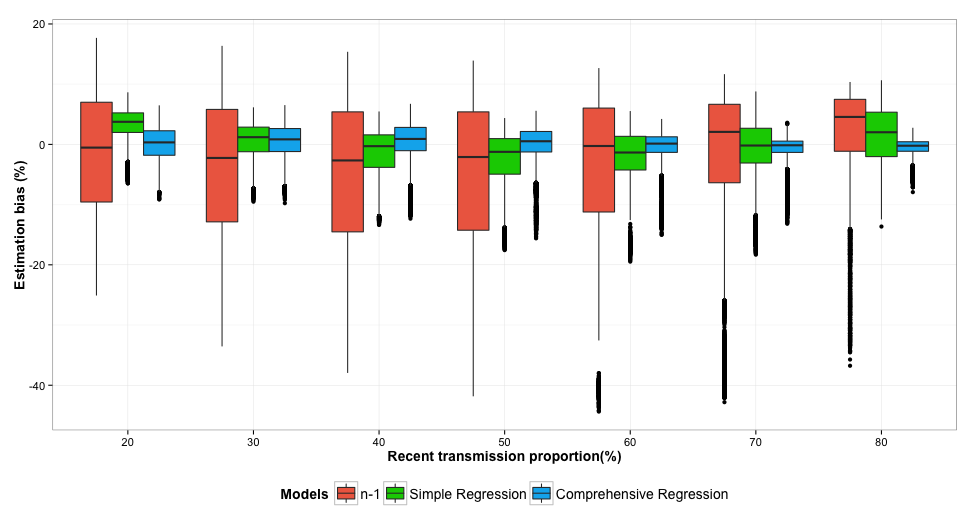


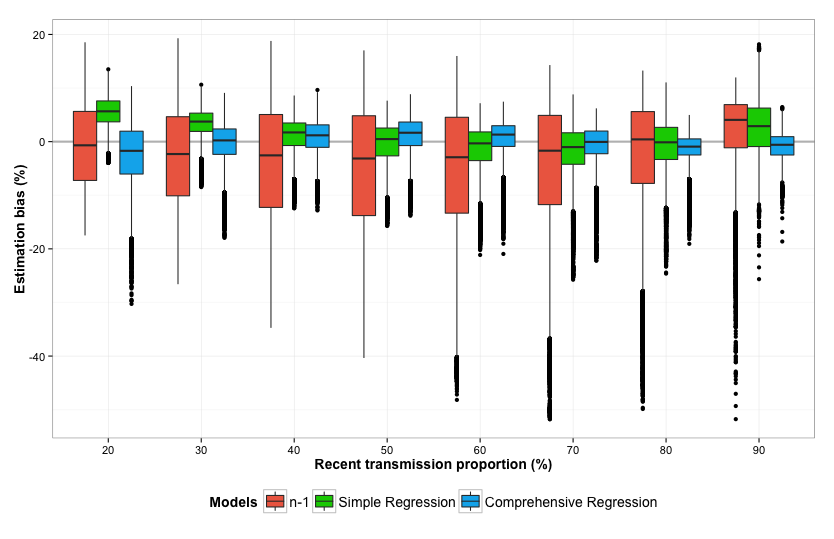


Figure H: Bias in estimates of the TB recent transmission proportion, comparing the ‘*n*-1’ method to the regression-based models in the derivation set (top) and validation set (bottom). The x-axis denotes the recent transmission proportion (simulated) in each set of simulations. The y-axis presents the percent estimation bias [(estimated value – true value) × 100] of the proportion of incident active TB due to recent transmission. Estimates from the ‘n-1’ method are shown in red, and those from the simple- and comprehensive- regression tools are shown in green and blue respectively. Boxes show the interquartile range of values from all simulations, and “whiskers” show the 95% confidence intervals, such that narrower boxes correspond to more precise (reproducible) estimates.

Table C compares the absolute bias (absolute value of true minus estimated proportion) in different estimation methods for the recent transmission proportion, comparing the derivation and validation sets of scenarios.

Table C: Absolute estimation bias.

|  | **Absolute Bias (%)** | | | | |  |
| --- | --- | --- | --- | --- | --- | --- |
| **Derivation set** | **1st Qu.** | **Median** | **Mean** | **3rd Qu.** | **Max** | |
| ‘*n-*1’ | 3.93 | 7.78 | 9.09 | 12.38 | 44.37 | |
| Simple Regression | 1.36 | 2.73 | 3.36 | 4.66 | 19.47 | |
| Comprehensive Regression | 0.68 | 1.57 | 2.05 | 3.05 | 15.6 | |
| **Validation set:** |  |  |  |  |  | |
| ‘*n-*1’ | 3.65 | 7.47 | 8.9 | 12.2 | 51.8 | |
| Simple Regression | 1.56 | 3.12 | 3.5 | 4.91 | 25.7 | |
| Comprehensive Regression | 1.08 | 2.3 | 2.72 | 3.82 | 30.3 | |

## Partial rank correlations

Analysis of partial rank correlation coefficients (PRCC), in conjunction with Latin hypercube sampling, is an efficient tool for uncertainty analysis of models with a large number of factors [9]. Using the residuals obtained from the regression procedure, partial correlation characterizes the linear relationship between a specific model parameter (*x_i_*) and an outcome measure (*y*) after discounting the linear effect of other model parameters (*x_j_ ≠ x_i_*). Analysis of partial correlation coefficients is a robust sensitivity measure for nonlinear but monotonic relationships between inputs and outputs, as long as little or no correlation exists between the inputs. Compared to an ordinary partial correlation coefficient procedure, PRCC is considered more powerful at determining the sensitivity of a parameter that is strongly monotonic yet highly nonlinear, by performing a rank transformation.

Table D shows the partial rank correlations for the estimation bias in each model with regard to various epidemiological/study characteristics. All values are statistically significant with a p-value of < 0.0001. While the assumptions of inputs’ independence (e.g., *c* and *n* are highly correlated), and monotonic relationships between inputs and outputs (e.g., non*-*monotonic relationship of ‘*n-*1’ bias with incidence) are not strictly met, the results can still provide insight on existing relationships between model outputs and regression parameters.

The bias in the ‘*n-1*’ estimates of the recent transmission proportion were strongly and negatively correlated with the number of clusters, and positively correlated with the duration of the study and with the coverage of fingerprinting (Table D). Thus, in short studies where the sampling coverage was incomplete, the ‘*n-*1’ method could be expected to give estimates of the recent transmission proportion that are much lower than the actual value. Both regression methods provided estimates that were much less closely correlated to these variables. By contrast, the regression*-*based estimates were somewhat more strongly (and negatively) correlated with TB incidence, such that as incidence increased (providing little additional room for positively-biased estimates), the regression tools tended to underestimate the recent transmission proportion.

Table D: Partial rank correlation between the bias in estimates of the recent TB transmission proportion and epidemiological/study characteristics.

|  | **Partial rank correlation coefficient**^[[4]](#footnote-4)^ | | | | |
| --- | --- | --- | --- | --- | --- |
|  | **Proportion of observed clustered cases in sample (*c*)** | **Proportion of observed clusters in sample (*n*)** | **Sampling duration (*d*)** | **Sampling coverage (*p*)** | **Disease incidence (*i*)** |
| **Estimation bias** |  |  |  |  |  |
| ‘*n-*1’ | 0.281 | -0.211 | 0.792 | 0.889 | -0.073 |
| Simple regression | -0.153 | 0.003 | -0.03 | 0.165 | -0.185 |
| Comprehensive regression | 0.082 | 0.101 | -0.226 | -0.066 | 0.02 |

## Comparison to illustrative data from Malawi

We tested the performance of our regression tool in a large-scale whole genome sequencing study in the Karonga district of Malawi [11]. This study includes 1687 samples of TB patients gathered from 1995 to 2010. The authors define a case of tuberculosis *“as being due to recent infection if a source case was identified in the network within the previous 5 years, and not being due to recent infection if no source was identified or if the closest source (in terms of number of SNPs) was more than 5 years earlier.*” They subsequently estimate that 38% of patients had evidence of recent infection.

We used the study characteristics to estimate the input parameters to the simple regression model (Table E), which estimated the recent transmission proportion at 32%. This estimate is consistent with the published estimate (using the “n-1” approach, but within a 5-year window) of 38%.

**Table E: Regression model input values.**

| Inputs | Value | Description | Computational step |
| --- | --- | --- | --- |
| *SS* | 1687 | Sample size | Reported |
| *C* | 1113 | Number of clustered cases in the sample | Reported |
| *N* | 717 | Number of observed clusters in the sample | Manually counted via reported figures in the manuscript |
| *P* | 0.41 | Proportion of cases sampled | Computed as the proportion of expected incident TB cases over 15 years (given reported TB incidence in Karonga) for which a DNA sample is available |
| *D* | 15 | Duration of study | Reported |
| *I* | 100 | Underlying incidence | 2009 estimate |

# Sensitivity Analysis

## Fingerprint replacement rate

We studied the sensitivity of results to variation of the fingerprint replacement rate parameter. We changed the underlying replacement rate to half (0.05) and double (0.2) the original value, and compared the performance of the regression models with the baseline scenario (0.1). Figure I shows the subsequent effect of the replacement rate on the average ratio of circulating strains in the model.


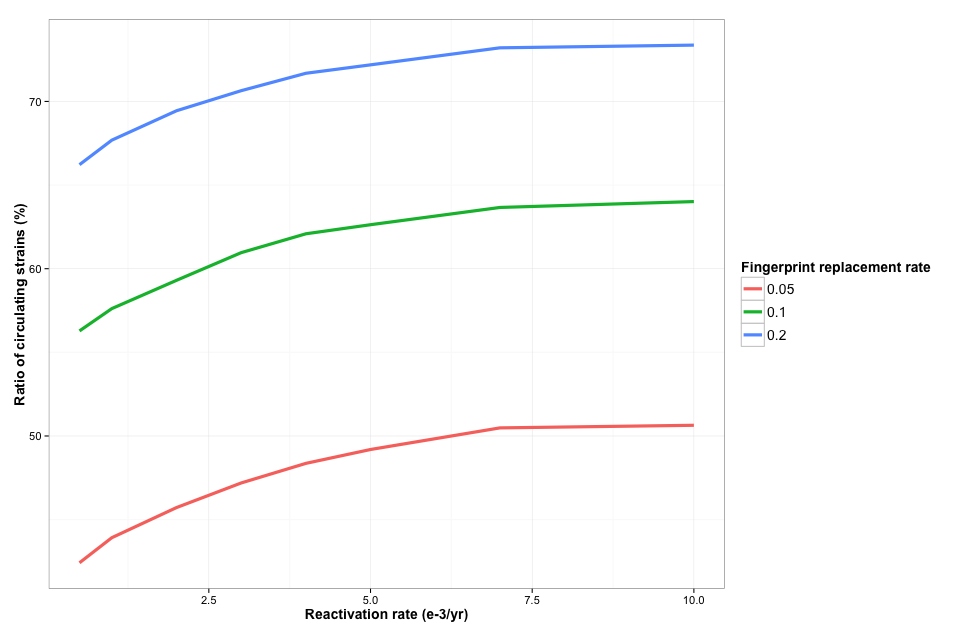


Figure I: Effect of the fingerprint replacement rate on the ratio of circulating strains (RCS) in the simulated population. As also seen in Figure 1, increasing the fingerprint replacement rate results in an increase in the ratio of circulating strains, for any given level of replacement.

Using an experimental setting for DNA sampling and analysis similar to that used in the derivation set, we compared the effect of replacement-rate variation on the regression models’ performance (Figure J). The results suggest a similar pattern in the estimators’ behaviour: at higher levels of the fingerprint replacement rate, the models tend to underestimate the recent transmission proportion, visible through an overall shift toward more negative bias values in Figure J. This can be explained as the effect of a higher ratio of circulating strains resulting in a more disperse (left-skewed) distribution of clusters. Such left-skewed distributions entail a large number of small-size clusters that are easier to be missed by incomplete samples of the population, thus inducing an underestimation of the true recent transmission proportion. Note that this effect is seen for all estimation methods (both ‘n-1’ and regression). In all scenarios, the regression models provide a more accurate estimation method (with smaller range). Given that the precision of estimates is somewhat lower in settings with lower replacement rates (i.e., wider bars on the left-hand side of Figure J), more representative genotyping data on the diversity of TB strains in high-burden settings would be useful to better calibrate the underlying replacement-rate value and thereby improve the regression models’ estimates.


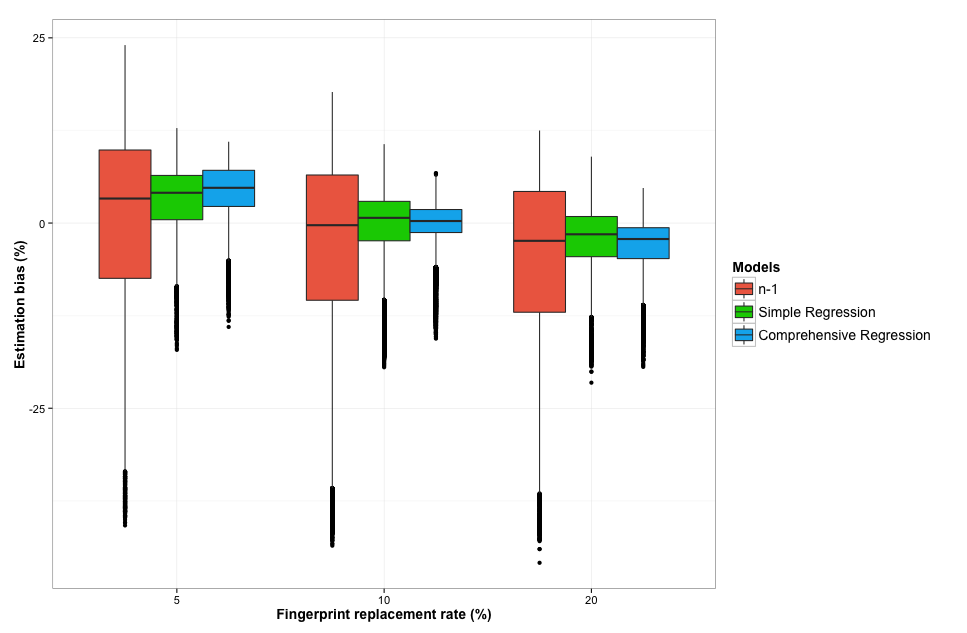


Figure J: Bias in different methods for estimating the recent TB transmission proportion among scenarios with different fingerprint replacement rates.

Variation in the underlying DNA fingerprint-replacement rate for latent individuals did not show significant influence on the results. Increasing the replacement rate resulted in a higher diversity of circulating strains, providing a right-skewed distribution of cluster sizes (including a large number of small-size clusters), and therefore increasing the likelihood of underestimating recent transmission proportion due to incomplete sampling.

## Sensitivity analysis to evaluate model performance in very high incidence settings

We studied the performance of the regression models in the 419 simulated scenarios with an incidence greater than 450 per 100,000/year that were originally excluded from the validation set. Of note, the current design does not provide a uniform sample of such scenarios (Figure K, left panel), and the results may not be precise for those few scenarios with incidence greater than 1300 per 100,000/year. Figure L compares the performance of the different models at various levels of incidence above the 450 per 100,000/year threshold. While such high-burden settings were not included in the original set of scenarios (the derivation set) used to derive the regression models, the narrower boxes in this figure indicate that the regression models may still provide less biased estimates of the recent transmission proportion than the ‘*n-*1’ method, at least in settings with incidence lower than 1300 per 100,000/year.


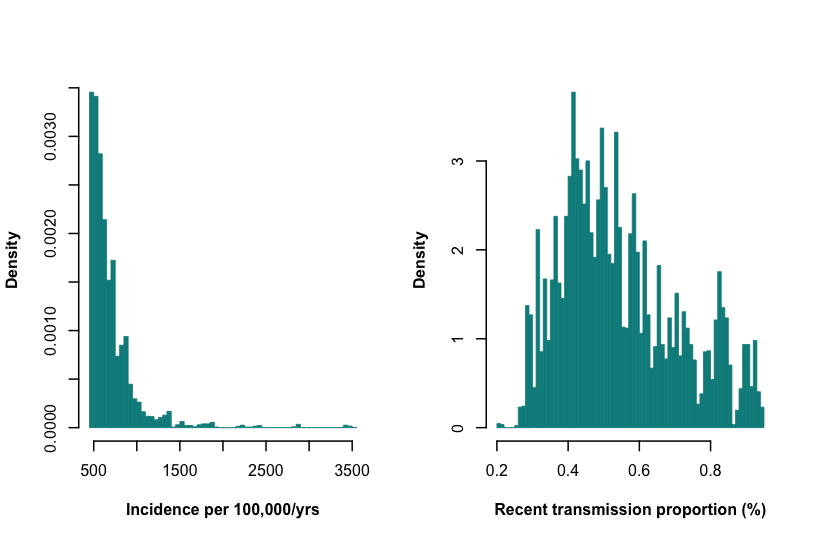


Figure K: Distribution of average incidence and recent transmission proportions in high-burden TB scenarios.


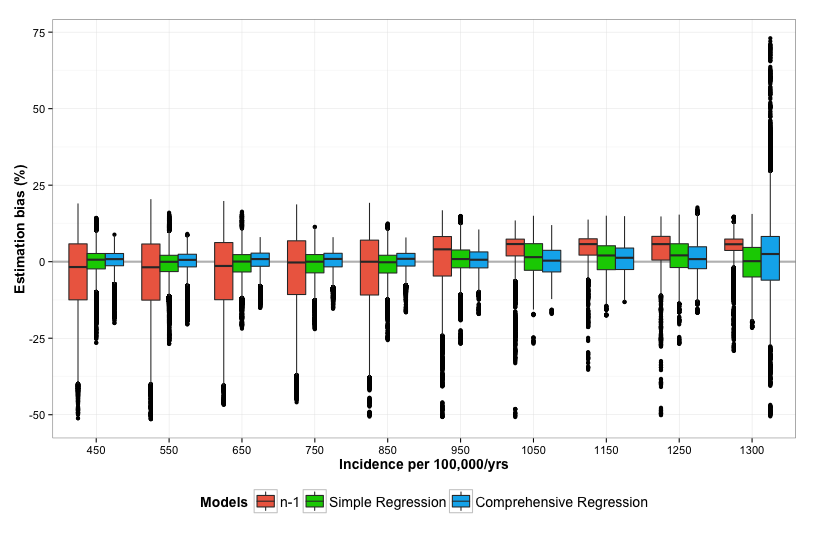


Figure L: Estimation bias for recent transmission proportion, comparing the ‘*n-*1’ method to the regressio*n-*based models in high-burden scenarios. Due to the small sample, all scenarios with incidence greater than 1300 are included together (at the far right side of the x-axis).

# Illustrative case study

To investigate the potential performance of the regression tools in a real setting, we benchmark a representative study from the TB genotyping literature and calibrate the simulation model to represent the corresponding epidemiological settings and study characteristics. For this purpose, we adopt [10], a study performed in Cape Town, South Africa, from 1993 to 1998. The authors analyzed DNA fingerprints of 765 TB patients during this period and estimated the proportion of recent disease transmission that occurs in households (as opposed to in the community).

We simulated a population of 38,600 individuals corresponding to the potential catchment area of the published study [10]. To estimate the underlying TB incidence, we considered two scenarios with regard to the case detection ratio: scenario 1 using a high case detection ratio of 90%, and scenario 2 using the current estimate of 62% for South Africa. (See the main manuscript text for further details.) In each scenario, we then computed the local incidence and estimated the DNA sample coverage respectively. Table F summarizes the list of variables and the calculation steps for deriving each value.

Table F: List of parameters in each scenario.

| **Parameters** | **Value** | | **Source/description** |
| --- | --- | --- | --- |
|  | **Scenario 1** | **Scenario 2** |  |
| Population size | 38600 | | [10] |
| Total number of patients with incident active TB over the study period (*N*) | 1094 | |  |
| Patients with available DNA sample (*SS*) | 765 | |  |
| Study duration (*d*) | 6 | |  |
| Proportion of clustered cases in sample (*c*) | 0.72 | |  |
| Proportion of clusters in sample (*n*) | 0.16 | |  |
| Proportion of detected cases started on treatment (*b*) | 86% | | Simulated [12] |
| Case detection ratio (*a*) | 90% | 62% | Assumption |
| Annual local incidence (*i*) | 203 | 294 | *N/(a*d)* |
| Sample coverage for simulation (*p*) | 0.73 | 0.50 | *SS/ (i * d * b)* |

In each scenario, we initially fixed the study characteristics (e.g., duration of six years and coverage of 73% for scenario 1), and calibrated the contact rate and annual replacement rate parameters to provide the specified incidence level (e.g., 202 cases) and observed levels of clustering in the simulated samples (e.g., *n*=16%, *c*=72%). Once the simulations were calibrated, we carried out a series of data-collection exercises for various levels of sampling duration and coverage, and compared the estimated levels of recent transmission proportion through the ‘*n-*1’ model and regression-based models against the true (simulated) level of recent transmission.

Figure M (Figure 4 in the main manuscript) and S15 illustrate one-way sensitivity analyses of estimation bias across varying levels of study coverage (fixing duration at 6 years per original study), and study duration (fixing coverage at 73% per original study) in each scenario. In the original study, the ‘*n*-1’ method provides a close estimate of the underlying recent transmission proportion (<1% bias), which owes to complete sample characteristics (e.g., sample duration of 6 years and coverage of 73% in scenario 1). This performance, however, deteriorates for settings with limited availability of fingerprinting data. At low levels of sampling duration and coverage, the ‘*n-*1’ model underestimates the true level of recent transmission proportion by a great margin (10% to 20%), while the regression-based models provide more accurate and precise estimates of this value (5% to 10%). The performance of ‘*n*-1’ method further deteriorates in scenario 2 due to the assumption of lower underlying case detection and therefor lower sampling coverage estimated at 50%.


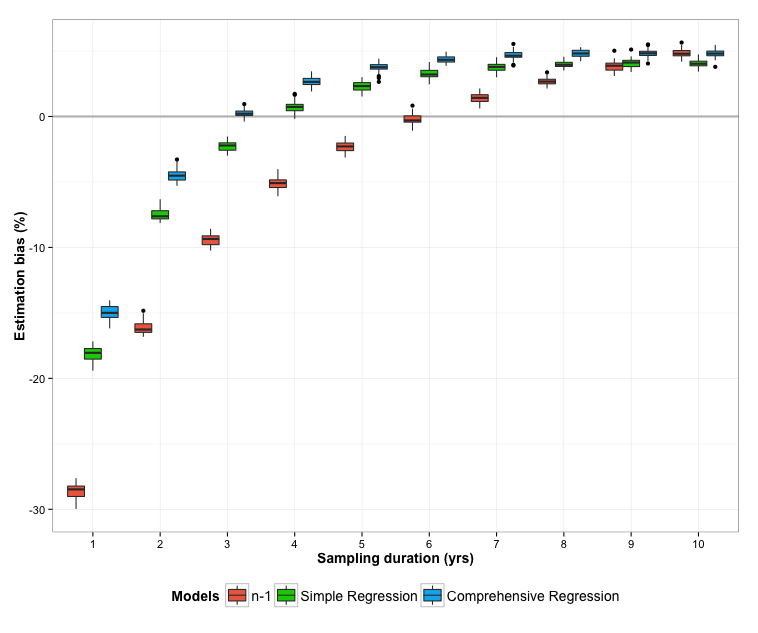

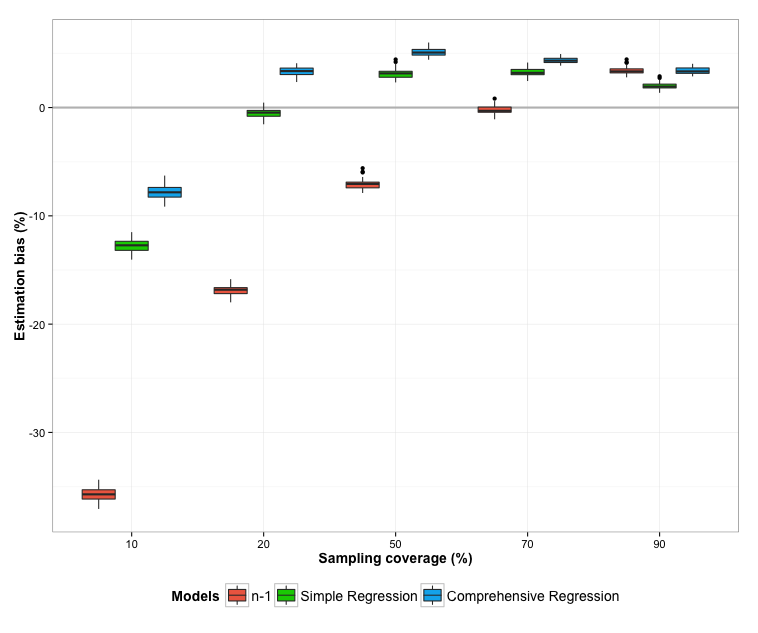


*

*

Figure M: One-way sensitivity analysis of estimation bias for TB recent transmission proportion in scenario 1. Panel A compares the estimation bias resulting from each model at a fixed study of 73% and for various levels of study duration. Panel B present the results at a fixed study duration of six years and for various levels of study coverage. The shaded area marks the 10% threshold for acceptable accuracy of estimated values. * marked values denote the study settings in [10].


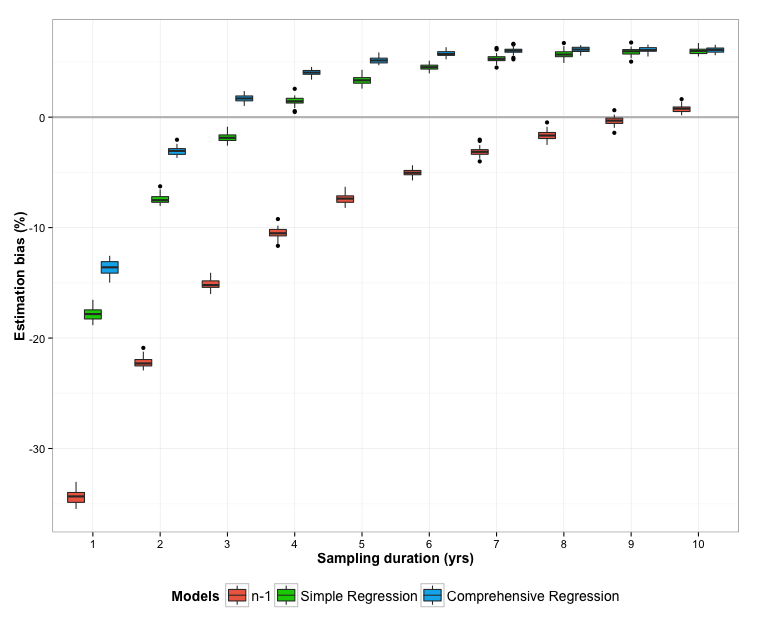

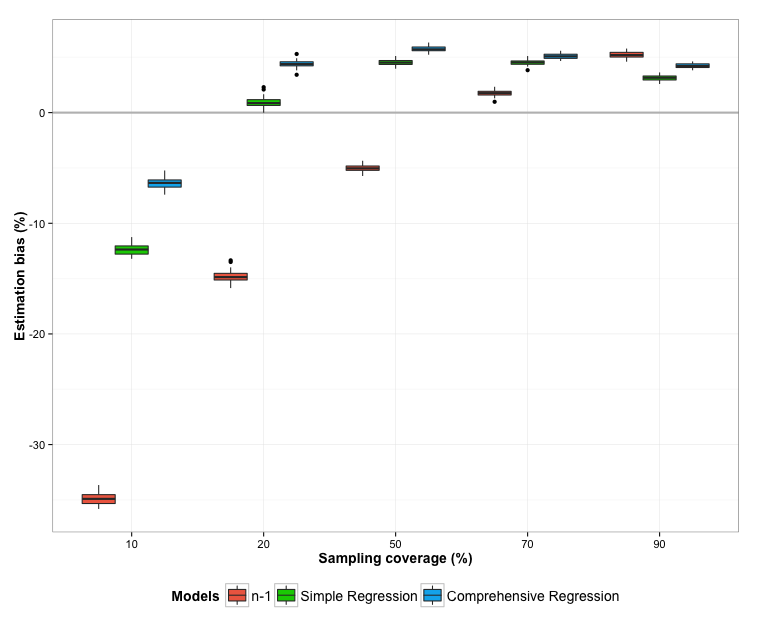


*

*

Figure N: One-way sensitivity analysis of estimation bias for the TB recent transmission proportion in scenario 2. Panel A compares the estimation bias resulting from each model at a fixed study of 50% and for various levels of study duration. Panel B present the results at a fixed study duration of six years and for various levels of study coverage. The shaded area marks the 10% threshold for acceptable accuracy of estimated values. * marked values denote the study settings in [10].

**References**

1. Vynnycky E, Fine PE. The natural history of tuberculosis: the implications of age-dependent risks of disease and the role of reinfection. Epidemiol Infect. 1997;119: 183–201.

2. Kasaie P, Andrews JR, Kelton WD, Dowdy DW. Timing of tuberculosis transmission and the population-level impact of household contact tracing: an agent-based simulation model. Am J Respir Crit Care Med. 2014;189: 845–852.

3. World Health Organization. Global TB Report [Internet]. 2013. Available: http://www.who.int/tb/publications/global_report/en/

4. Kasaie P, Kelton WD, Dowdy DW. Estimating proportion of tuberculosis recent transmission via simulation. In: Tolk A, Diallo SD, Ryzhov IO, Yilmaz L, Buckley S, Miller JA, editors. Proceedings of Winter Simulation Conference,IEEE. Piscataway, New Jersey: Institute of Electrical and Electronics Engineers, Inc.; 2014. pp. 1469–1480.

5. Murray M. Determinants of cluster distribution in the molecular epidemiology of tuberculosis. Proc Natl Acad Sci. 2002;99: 1538–1543.

6. Fok, A. YN, Schulzer M, FitzGerald MJ. Risk factors for clustering of tuberculosis cases: a systematic review of population-based molecular epidemiology studies [Review Article]. Int J Tuberc Lung Dis. 2008;12: 480–492.

7. Houben RM, Glynn JR. A systematic review and meta‐analysis of molecular epidemiological studies of tuberculosis: development of a new tool to aid interpretation. Trop Med Int Heal. 2009;14: 892–909.

8. Moonan PK, Ghosh S, Oeltmann JE, Kammerer JS, Cowan LS, Navin TR. Using genotyping and geospatial scanning to estimate recent mycobacterium tuberculosis transmission, United States. Emerg Infect Dis. 2012;18: 458–465.

9. Sanchez MA, Blower SM. Uncertainty and sensitivity analysis of the basic reproductive rate: tuberculosis as an example. Am J Epidemiol. 1997;145: 1127–1137.

10. Verver S, Warren RM, Munch Z, Richardson M, van der Spuy GD, Borgdorff MW, et al. Proportion of tuberculosis transmission that takes place in households in a high-incidence area. Lancet. 2004;363: 212–4. doi:10.1016/S0140-6736(03)15332-9

11. Guerra-Assunção JA, Crampin AC, Houben RMGJ, Mzembe T, Mallard K, Coll F. Large-scale whole genome sequencing of M. tuberculosis provides insights into transmission in a high prevalence area. Elife. 2015;4: e05166.

12. MacPherson P, Houben RMGJ, Glynn JR, Corbett EL, Kranzer K. Pre-treatment loss to follow-up in tuberculosis patients in low- and lower-middle-income countries and high-burden countries: a systematic review and meta-analysis. Bull World Health Organ. World Health Organization; 2014;92: 126–38. doi:10.2471/BLT.13.124800

1. *x:y* denotes the interaction effect between factor *x* and *y*. [↑](#footnote-ref-1)
2. In a practical setting, the underlying level of TB incidence is often unknown, and information is restricted to the domain of available data from historical TB diagnosis rates. In such settings, the case detection ratio can also be used to determine the uncertain level of TB incidence, where (annual) incidence is estimated as the number of diagnosed TB cases (over a year) divided by the case detection ratio. [↑](#footnote-ref-2)
3. The simulation model assumes a high case detection ratio of 86% in the derivation set of scenarios, and models the sampling coverage as the likelihood of including these diagnosed cases in the study. In application of the simulation model, we therefore estimate the local study coverage with regard to this value. [↑](#footnote-ref-3)
4. More strongly positive numbers suggest that the model overestimates the recent transmission proportion at higher levels of the input value, whereas more strongly negative numbers suggest underestimation with higher input values. [↑](#footnote-ref-4)
